# Aerobic Damage to [FeFe]-Hydrogenases: Activation Barriers for the Chemical Attachment of O_2_**

**DOI:** 10.1002/anie.201400534

**Published:** 2014-03-11

**Authors:** Adam Kubas, David De Sancho, Robert B Best, Jochen Blumberger

**Affiliations:** Department of Physics and Astronomy, University College LondonGower Street, London WC1E 6BT (UK); Department of Chemistry, Cambridge UniversityLensfield Road, Cambridge CB2 1EW (UK); Laboratory of Chemical Physics, National Institute of Diabetes and Digestive and Kidney Diseases, National Institutes of HealthBethesda, MD 20892-0520 (USA)

**Keywords:** ab initio calculations, electron transfer, [FeFe]-hydrogenases, iron–sulfur clusters, oxygen activation

## Abstract

[FeFe]-hydrogenases are the best natural hydrogen-producing enzymes but their biotechnological exploitation is hampered by their extreme oxygen sensitivity. The free energy profile for the chemical attachment of O_2_ to the enzyme active site was investigated by using a range-separated density functional re-parametrized to reproduce high-level ab initio data. An activation free-energy barrier of 13 kcal mol^−1^ was obtained for chemical bond formation between the di-iron active site and O_2_, a value in good agreement with experimental inactivation rates. The oxygen binding can be viewed as an inner-sphere electron-transfer process that is strongly influenced by Coulombic interactions with the proximal cubane cluster and the protein environment. The implications of these results for future mutation studies with the aim of increasing the oxygen tolerance of this enzyme are discussed.

Hydrogenases are enzymes that catalyze hydrogen oxidation and reduction according to the reaction H_2_⇄2 H^+^+2 e^−^.[1] Among the various members of this enzyme family, [FeFe]-hydrogenases show the highest activity for hydrogen production, with a turnover rate constant of over 10 000 s^−1^.[2] Although synthetic catalysts with even higher turnover rates have recently been reported,[3] hydrogenases are still unrivalled in terms of reversibility and the low overpotential required. They have been successfully used as catalysts in biofuel cells in the laboratory,[4] but applications at an industrial scale are hampered in part by the irreversible inhibition and damage of [FeFe]-hydrogenases by molecular oxygen.[5] Some membrane-bound [NiFe]-hydrogenases[6] can operate in the presence of oxygen but they are biased towards hydrogen oxidation.[7] Hence, there are currently intense efforts ongoing to make [FeFe]-hydrogenase, the best H_2_ producers, less sensitive to O_2_.[2a, 8]

Early electrochemical studies have indicated that oxygen inhibition of [FeFe]-hydrogenases proceeds through the reversible formation of an oxygen adduct and is followed by slower irreversible damage to the enzyme.[9] Later, evidence was reported that the destruction of the enzyme is initiated by O_2_ binding to one of the Fe atoms of the di-iron cluster, where it is converted to a reactive oxygen species (ROS) that subsequently destroys the neighbouring [Fe_4_S_4_] cubane cluster (Figure 1).[10] This picture is consistent with the theoretical work of Reiher et al.,[11] who performed extensive density functional theory (DFT) studies on the regioselectivity of oxygen binding to [FeFe]-hydrogenses and on possible reaction intermediates.

**Figure 1:**
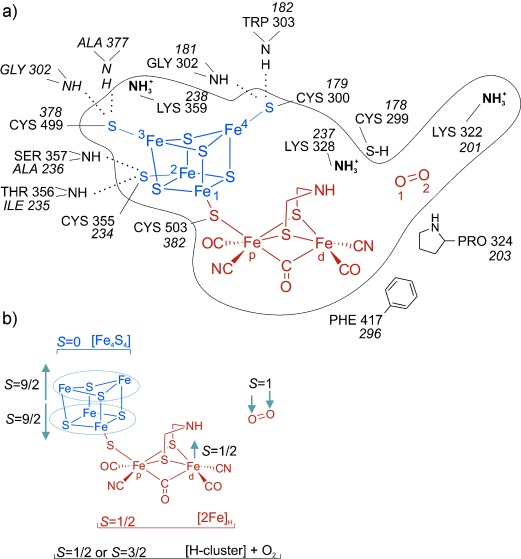
The active site of the [FeFe]-hydrogenases. The H-cluster is shown in blue and red, along with interacting triplet oxygen. a) the protein binding pocket of the H-cluster with all of the residues included in the large model. Amino acid abbreviations and numbers are given for *Cp* and *Dd* in regular and italic font, respectively. In the ONIOM approach, the high level was chosen as all atoms inside the closed figure. b) The spin-coupling scheme adopted in this study.

In addition to the thermodynamics of O_2_ binding studied in the above-mentioned works, it is crucial to understand the kinetics of this process. Overall O_2_ and CO binding rates (also termed inactivation rates *k*_in_) have recently been measured.[2c, 12] However, it remains unclear whether these rates are limited by actual chemical bond formation between O_2_ and the di-iron site or by diffusion of O_2_ from the solvent to the active-site pocket.[13] Another alternative is that both steps occur on the same time scale as was indeed found for CO_2_ binding to carbon monoxide dehydrogenase/acetyl-CoA synthase.[14] This knowledge is important because any attempt to reduce oxygen binding would be most effective if the slowest step is targeted.

Hence, a first step and the focus of the current work is to obtain reliable estimates for the activation barrier for the chemical attachment of O_2_ to the di-iron site. This is challenging because of the complicated electronic structure of the hydrogenase active site. Herein, we address this problem through re-parameterization of a range-separated hybrid density functional against correlated ab initio reference data at the *n*-electron valence state perturbation theory (NEVPT2)[15] level. The estimated activation free energy, 13 kcal mol^−1^ for [FeFe]-hydrogenases from *Clostridium pasteurianum* (*Cp*)[16] and *Desulfovibrio desulfuricans* (*Dd*),[17] suggests that chemical bond formation constitutes a major barrier for the O_2_ binding process. The calculations reveal that spontaneous electron transfer (ET) from the di-iron site to O_2_ makes the oxygen attachment thermodynamically favorable, thus suggesting that mutations that counteract this ET may help to increase oxygen resistance.

We have investigated a large computational model for the enzyme active site (Figure 1 a). It contains the di-iron site (red) and the adjacent [Fe_4_S_4_] cluster (blue), collectively referred to as the H-cluster, as well as all close-lying residues that form hydrogen bonds with atoms of the H-cluster. The models for the *Dd* (PDB id: 3C8Y) and *Cp* (PDB id: 1HFE) enzymes are almost the same except that Gly 302 and Ala 377 are included only for the *Dd* protein because they do not form hydrogen bonds in the *Cp* structure. Calculations were carried out for the catalytically active H_ox_ state, which has been shown to be the relevant state for oxygen inhibition[10] (Figure 1 b, see the Experimental Section for further details).

Geometry optimization of this large model with the BP86 functional gives very good agreement with the crystal structure (Table S3 in the Supporting Information) in line with the good performance reported previously.[18] However, it can be expected that the electron delocalization error of this density functional[19] significantly affects the activation barrier for oxygen binding. We investigated this issue in more detail and constructed an active-site model (indicated in red in Figure 1, CYS 503/382 was replaced with CH_3_SH) that is small enough to permit reference calculations at the NEVPT2 level of theory. The O_2_ binding curves obtained are shown in Figure 2. An energy barrier of 5.6 kcal mol^−1^ and a binding energy of −7.6 kcal mol^−1^ were obtained at the NEVPT2 level. With the BP86 functional, the potential energy curve is purely attractive and the binding energy is overestimated by 2.5 kcal mol^−1^. Global hybrid functionals such as B3LYP predict a very small barrier at the cost of significantly reduced binding energy. This observation prompted us to parametrize a range-separated hybrid functional based on CAM-B3LYP[20] with modified parameters *α*=0.1, *β*=0.9, *μ*=0.5 (10 % Hartree Fock exchange at *r*_12_→0 and 100 % at *r*_12_→∞; *r*_12_ is the interelectronic distance) to yield a best fit to the NEVPT2 data. The corresponding functional is denoted CA1-B3LYP. It captures both the activation barrier (6.4 kcal mol^−1^) and the binding energy (−5.1 kcal mol^−1^) to good accuracy (Figure 2), as well as other properties such as weak interactions and hydrogen bonding (see Table S1 in the Supporting Information).

**Figure 2:**
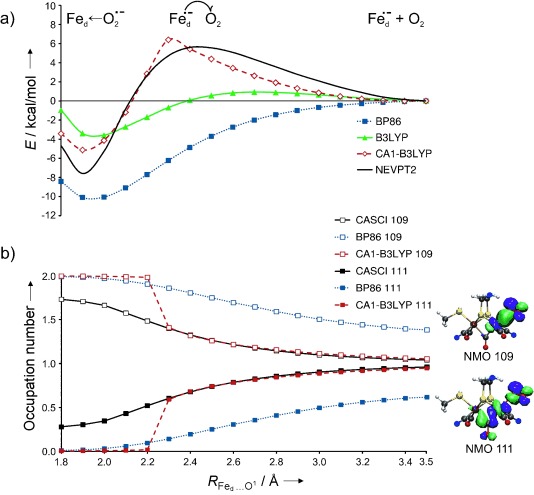
a) The relative energy change upon oxygen binding to the distal iron atom of the small cluster calculated by various methods. b) The change in the occupation number of the natural molecular orbitals corresponding to σ and σ* bonds between the distal iron and the oxygen molecule. Details can be found in the Supporting Information.

An explanation for why our range-separated functional works can be found in Figure 2 b. At long distance, both the binding and anti-binding combinations of 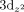 (Fe_d_) and π* (O_2_) orbitals represented by natural molecular orbitals (NMOs) 109 and 111, respectively, have occupation numbers of one. This picture is obtained with the reference calculations and with our new functional but not with BP86. In the latter case, spurious charge-transfer can be seen at all distances. The CA1-B3LYP functional follows the reference curve up to the distance of 2.3 Å, where a switch from one potential energy surface (Fe_d_^−^⋅⋅⋅O_2_) to another occurs (Fe_d_←O_2_^−^) that can be interpreted as electron transfer from Fe_d_ to O_2_. Thus, the variable amount of exact exchange in the “single-reference” density functional is able to mimic the two important charge transfer configurations.

The CA1-B3LYP functional was subsequently used to calculate the activation energy (Δ*E*^≠^) and binding energy (Δ*E*) for the large model (solid lines in Figure 3). A potential energy maximum was found at an Fe_d_⋅⋅⋅O^1^ interatomic distance of 2.4 Å for both enzymes, a value similar to that in the small model. In the case of *Cp*, the binding energy (−17.0 kcal mol^−1^) is lower by 2 kcal mol^−1^ compared to the *Dd* enzyme, while the electronic barriers are within the error of the present calculations (3.0 kcal mol^−1^ and 3.6 kcal mol^−1^ for *Cp* and *Dd*, respectively). The differences in binding energy are probably a consequence of the two additional residues that form hydrogen bonds to the cubane cluster in the *Dd* enzyme (Gly302 and Ala377), which lead to a slight reduction in the stabilizing electron donation from the cubane to the partially oxidized di-iron site.

**Figure 3:**
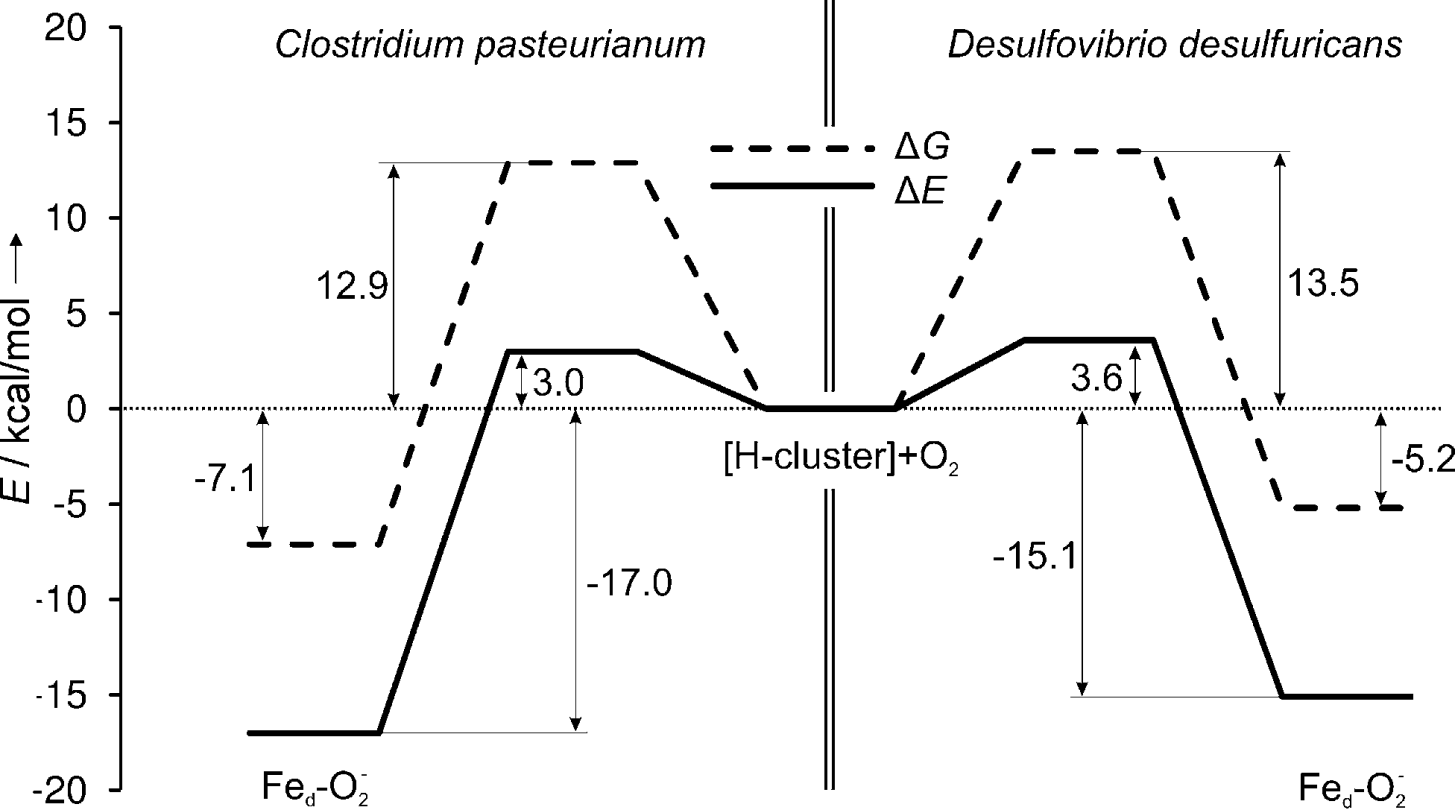
The relative energy change upon oxygen binding to H-clusters buried in the *Clostridium pasteurianum* (left) and *Desulfovibrio desulfuricans* (right) proteins. Solid and dashed lines represent the change in electronic energies and Gibb’s free energies, respectively.

Entropic effects are expected to be very large for binding processes owing to the loss of translational and rotational degrees of freedom of the ligand.[21] We have estimated the total entropic free energy contribution to be –TΔ*S*=10.4 kcal mol^−1^ (see the Supporting Information). On adding zero-point vibrational energy (1.5 kcal/mol) and thermal corrections (-1.0 kcal/mol), we obtain a contribution of 9.9 kcal mol^−1^ that needs to be added to the potential energies to obtain free energy differences. This shifts the activation barrier up to 13 kcal mol^−1^ and the binding energy to −5 to −7 kcal mol^−1^ (dashed lines in Figure 3).

The free energies obtained allow us to make a first direct comparison with the experimental rates of oxygen inactivation (see the Supporting Information). Converting the computed free energies into rates by using transition state theory and adopting diffusion rates from previous MD simulations for [NiFe]-hydrogenase,[22] we obtained values for *k*_in_ of 3.6 s^−1^ mm^−1^ and 1.2 s^−1^ mm^−1^ for the *Cp* and *Dd* enzymes, respectively. The agreement with the experimental values (2.5 s^−1^ mm^−1^ and 40 s^−1^ mm^−1^, respectively)[2c] is encouraging, especially in the case of *Cp*. This gives further credence to our re-parametrized functional because it shows that the dramatic changes to the electronic structure of the H-cluster upon oxygen binding are rather well described. To explain the difference in the observed rates for the two enzymes quantitatively, more accurate values for the diffusion rates would be required, which is beyond the scope of this communication.

Further calculations on additional active-site models suggest that the presence of the highly negatively charged [Fe_4_S_4_] cubane cluster plays an important role in oxygen binding (see the Supporting Information). This cluster stabilizes the di-iron site, which becomes partially oxidized through donation of electron density upon O_2_ attachment. The cubane cluster thus facilitates ET from Fe_d_ to O_2_, most probably through purely electrostatic interactions. The small difference we observed between the *Cp* and *Dd* protein models, which differed only in the number of hydrogen bonds donated to the cubane cluster, further supports this hypothesis. Therefore, the mutation of residues in the neighborhood of the cubane cluster into positively charged amino acids such as lysine should stabilize the negative charge on the cubane and make O_2_ binding less favorable. Of course, this perturbation should not be too large so that the impact on catalytic activity remains reasonably small. On this basis we suggest that possible target sites for future mutation studies could include Ser357 or Thr356 (Ala236 or Ile235 for *Dd*), all of which are nonconserved and in close proximity to the cubane cluster.

## Experimental Section

The lowest-energy spin coupling defined in Figure 1 b was chosen consistently with previous calculations.[11], [23] Although the location of the unpaired electron (on either Fe_p_ or Fe_d_) is the subject of ongoing discussions, all studies agree that both iron atoms are low-spin +I or +II.[24] We adopted the structure with a bridging CO molecule (Fe_p_^II^Fe_d_^I^ redox state).[24] In the DFT calculations, the broken-symmetry (BS) approach[25] was employed. The high negative charge of the H-cluster (−3) was compensated by three protonated proximal lysine residues to give a total charge of zero. We considered only the antiferromagnetic coupling with triplet oxygen (*S*_tot_=1/2) because at long distance, this state is essentially degenerate with the *S*=3/2 state.[26] Geometry optimizations were carried out with the BP86+D3 functional[27] and the def2-TZVP basis[28] as implemented in the Turbomole program.[29] CA1-B3LYP calculations on the BP86+D3 structures were carried out using the ONIOM approach[30] as implemented in the NWChem program.[31] The energy of the low-level layer (entire system) was evaluated with the BP86+D3 functional while the CA1-B3LYP functional was used for the high-level part encircled in Figure 1 a. NEVPT2 calculations were carried out using the ORCA 2.9.1 package.[32] Further details and Cartesian coordinates of all relevant structures can be found in the Supporting Information.
