# Supplementary material for: Aerobic Damage to [FeFe]-Hydrogenases: Activation Barriers for the Chemical Attachment of O2
Source: Angew Chem Int Ed Engl. 2014 Mar 11;53(16):4081–4. doi: 10.1002/anie.201400534 (PMC4143129; doi:10.1002/anie.201400534)
Supplement: Supplementary file 1 [file anie0053-4081-sd1.pdf]

Angewandte Chemie
Eine Zeitschrift der Gesellschaft Deutscher Chemiker

Supporting Information
© Wiley-VCH 2014
69451 Weinheim, Germany

Aerobic Damage to [FeFe]-Hydrogenases: Activation Barriers for the Chemical Attachment of O_2**

*Adam Kubas, David De Sancho, Robert B. Best, and Jochen Blumberger**

anie_201400534_sm_miscellaneous_information.pdf

Details of the QM calculations: geometry optimisation

All geometry optimisations were carried out with BP86 functional[1] and def2-TZVP basis[2] as implemented in Turbomole[3] program. Additionally, the empirical dispersion corrections in form proposed by Grimme were included[4] with the B-J dumpig scheme.[5] Resulting functional is denoted as BP86+D3 throughout the manuscript. Geometry optimisations were performed with the default settings (SCF convergence 10^{-6}, grid size m3). All single point energies were calculated with tightened SCF convergence (10^{-7}) and enlarged grid size ('m4' in the Turbomole nomenclature and 'fine' in NWChem). We took the advantage of the resolution of identity approximation[6] in all calculations. The activation was followed with constrained optimisations where $Fe_d \ldots O^1$ distance was varied between 3.5 Å to 1.8 Å and all C_α and nitrogen atoms in NH_3^+ groups of LYS 322 and 359 (*Cp* numbering, 201 and 238 in case of *Dd*) remained frozen.

Small model and CA1-B3LYP functional

The small model structure consist of an isolated $[2Fe]_H$ cluster where the $[Fe_4S_4]$ cubane was replaced with H^+ ion and bridging cysteine was substituted with a smaller CH_3-S^- fragment (see Figure S1). We followed the energy change with different methods in a series of single-point calculations on the top of optimised structures where the Fe…O distance was varied between 3.5 Å and 1.8 Å. In Figure S1 we show most the most important geometry changes that accompanies the oxygen binding. The oxygen-bound state was found at the Fe…O distance of 1.95 Å with O^1-O^2 bond length of 1.29 Å. Such lengthening of a bond between two oxygen atoms together with calculated vibration frequency of 1186 cm^{-1} indicates that the oxygen moiety is best described as a superoxo entity O_2^-.[7]

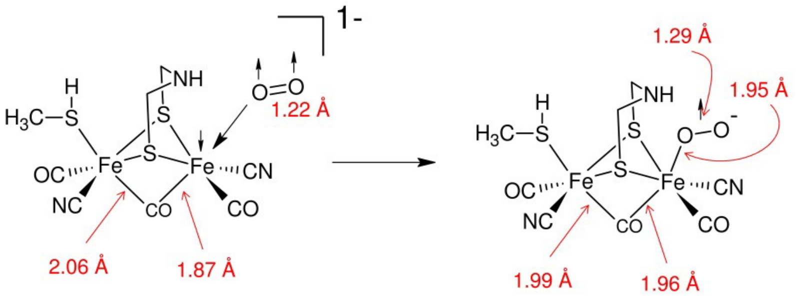

Figure S1. Geometry changes upon oxygen activation on the small cluster model.

The reference data were obtained with NEVPT2 method which base on the CASSCF reference wave function.[8] Because of higher basis-set demands of the wave function-based methods the def2-TZVPP basis set was employed which features additional polarization functions on Fe and S atoms. Although a full-valence CAS was not possible, ligands like CO/CN- create so strong ligand field that most of the doubly occupied and empty 3d orbitals of both iron atoms could remain inactive. We demonstrate this effect in Figure S2 where a qualitative scheme of orbital energies is presented. In fact, d_{z2} and d_{xz} orbitals of Fe_d, two π* orbitals of O_2 molecule and one π* orbital of the bridging CO along with five electrons [CAS(5,5)] were sufficient to describe the binding process (MOs enclosed in the teal rectangle in Figure S2; in Figure S3 the natural orbital isosurfaces are presented). In order to stabilise the active space over whole potential energy surface scan we employed state-averaging approach in which the CASSCF wave function was optimised for three doublet and two quartet states. Because selected active space can be considered as minimal, we performed additional MR-DDCI2 with small def2-SVP basis and we found that at each Fe…O distance the weight of the CASSCF reference was not smaller than 0.85 with other weights lower than 0.01.

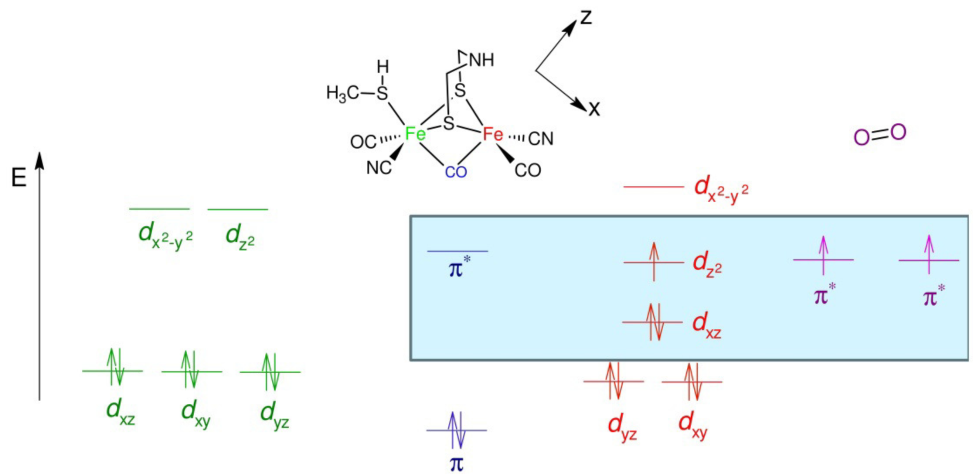

Figure S2. Qualitative molecular orbitals scheme of the small model system with active space orbitals marked in teal rectangle.

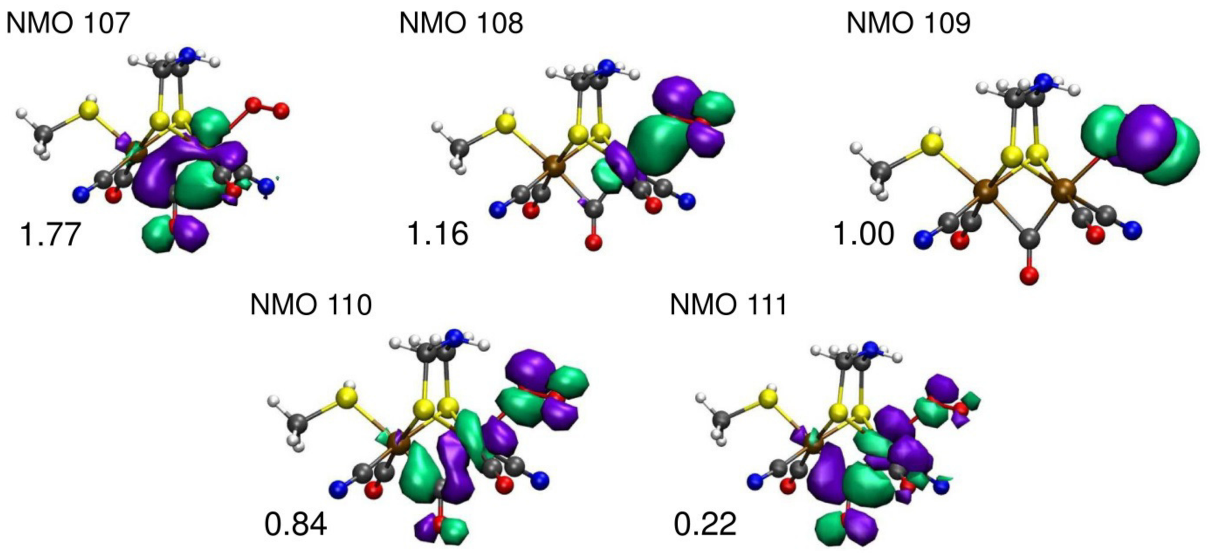

Figure S3. Isosurfaces of the natural orbitals obtained in the state-averaged CASSCF(5,5) calculations for Fe…O distance of 2.4 Å.

The reference calculations were compared with various functionals and in Figure S4a the one dimensional potential energy surface cut calculated with various DFT approaches is presented. Functionals tested include semi-local BP86, global hybrids B3LYP,[1a,9] BHandLYP[1a,b,d,8] with 20% and 50% of the Hartree-Fock exchange, respectively, and two range-separated (CAM-B3LYP,[10] LC-ωPBE[11]). The latter functionals base on the Ewald split of the Coulomb operator:[12]

$$\frac{1}{r_{12}} = \frac{1-[\alpha+\beta\cdot \mathrm{erf}(\mu r_{12})]}{r_{12}} + \frac{\alpha+\beta\cdot \mathrm{erf}(\mu r_{12})}{r_{12}} \quad (1)$$

where r_{12} is the interelectronic distance, erf is an error function and μ is range-separation parameter that is typically between 0.1 and 1. The parameter α defines a fixed amount of Hartree-Fock exchange (HFX) at all values of r_{12} while β regulates the variable amount. The sum of both these parameters give the maximum portion of HFX at long distance. The main differences between CAM-B3LYP and LC-ωPBE, apart from the form of the correlation part, are in the values of α, β and μ parameters which are $\alpha = 0.19$, $\beta = 0.46$, $\mu = 0.33$ for the former and $\alpha = 0.0$, $\beta = 1.0$, $\mu = 0.4$ for the latter.

In case of global hybrid functionals the barrier increases with the amount of HFX but the binding becomes less favourable. BHandLYP functional shows that the process is endothermic by 0.2 kcal/mol. Some improvement over B3LYP is its range-separated counter-part for which the barrier is 2.8 kcal/mol. Unfortunately, the error binding energy (3.9 kcal/mol) is still large. LC-ωPBE performes similar to BHandLYP. Thus we decided to tune the CAM-B3LYP functional to reproduce reference NEVPT2 activation barrier and binding

energy. To decrease the number of parameters we set $\alpha + \beta = 1$ so in the limit of infinite interelectronic separation the functional features 100% of HFX. We calculated the energies of bound state, transition state at Fe…O distance of 2.4 Å and complex at long Fe…O distance of 3.5 Å with α values between 0.0 and 0.4 and μ between 0.1 and 0.9. For each combination we calculated the relative error in ΔE and $\Delta E^{\ddagger}$ with respect to reference NEVPT2 data (-7.6 kcal/mol and 5.6 kcal/mol, respectively). In the next step, we took the sum of absolute values of these errors as a measure of total error. For each value of μ we computed the average of such total errors for all five α values between tested. Obtained data can be found in Figure S5. We see that for $\mu = 0.5$ a minimum has been found. The lowest error for this value of range-separation parameter was calculated for $\alpha = 0.1$. Thus the final optimised set of parameters are $\alpha = 0.1$, $\beta = 0.9$, $\mu = 0.5$. We also note that our relatively large range-separation parameter was reported to provide better activation barriers[13] what is now confirmed in our calculations. The new functional is denoted as CA1-B3LYP. We also carried out calculations on non-covalent complexation energies test set (NCCE31/04 test set of Zhao and Truhlar[14]) in order to evaluate the performance of CA1-B3LYP functional for interaction energies. The statistical evaluation can be found in Table S1. In most cases the new functional outperforms BP86 and B3LYP, especially for charge-transfer-dominated complexes. It performs surprisingly well for weak interactions as well what will be important for calculations on the large clusters. We note however that we set $\alpha + \beta$ to 1 and possible improvement is offered by an independent optimisation of all three parameters.

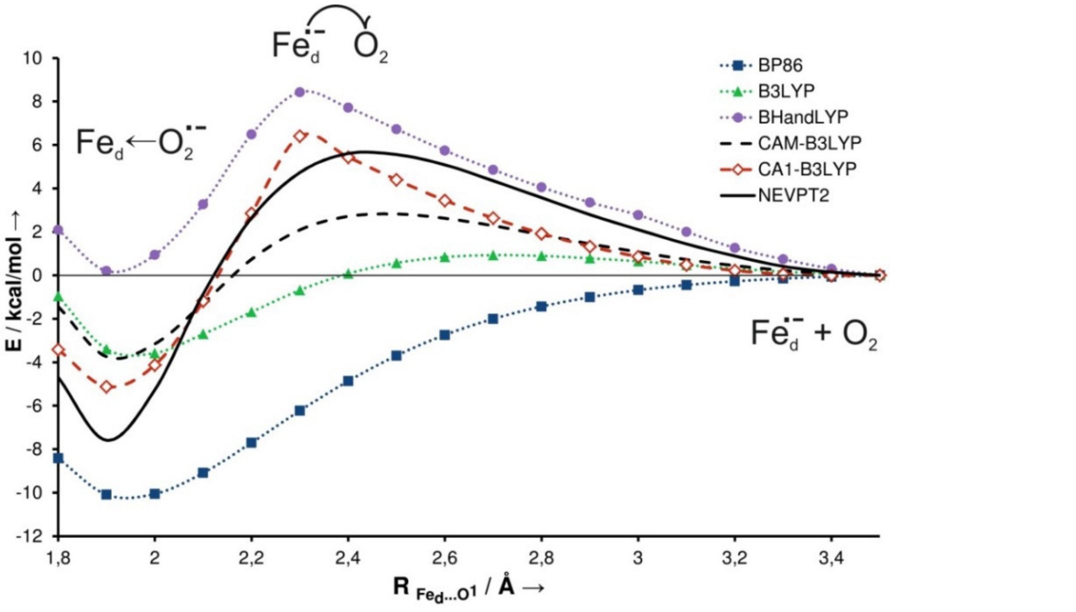

Figure S4. Relative energy change upon oxygen binding to the distal iron of the small cluster calculated with various methods. For each method energy at R = 3.5 Å is taken as a reference.

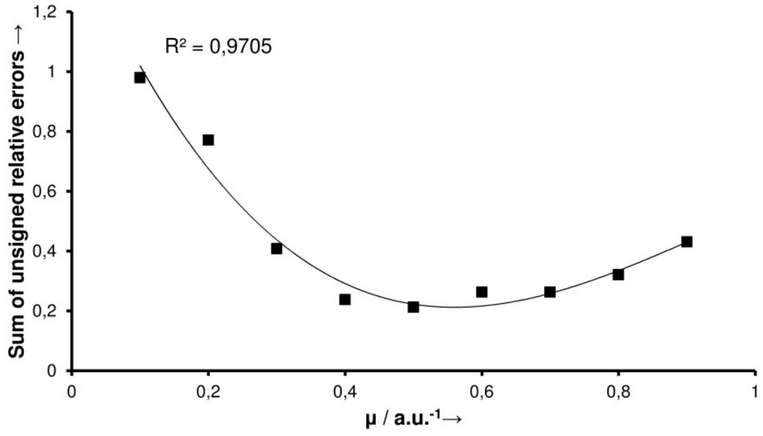

Figure S5. The dependency of the sum of unsigned relative errors with respect to range-separation parameter μ.

Table S1. Statistical evaluation of the NCCE31/04 test for BP86, B3LYP and CA1-B3LYP functionals. All values are in kcal/mol. Abbreviations HB6, CT7, DI6 and WI9 denote subsets of the reference database: hydrogen-bounded systems, charge-transfer dominated dimers, dipole-dipole interacting systems and weakly bound dimer, respectively.

	BP86	B3LYP	CA1-B3LYP
Mean Unsigned Error	1.09	0.65	0.52
Mean Squared Error	2.37	0.77	0.72
Mean Signed Error (MSE)	-0.05	-0.31	0.35
MSE – HB6	-0.72	-0.55	1.55
MSE – CT7	2.03	0.61	0.06
MSE – DI6	-0.54	-0.78	0.01

MSE – WI9	-0.90	-0.55	-0.01

The reference curve (black solid line) in Figure 2b was obtained with a doublet ground-state state-specific CAS configuration interaction (CASCI) wave function with the state-averaged CASSCF orbitals presented graphically on the right (orbital labels taken from Turbomole program).

Large cluster model and ONIOM calculations

The cluster models were constructed from X-Ray structures of *Cp* and *Dd* proteins (PDB codes: 3C8Y and 1HFE, respectively). Consistently with recent experimental and theoretical studies,[15,16] the di-μ-dithiolato bridge was modelled as $SCH_2(NH)CH_2S$. Residues included in the model are listed in Table S2. In both models all residues that can form a N-H…S(Cys) hydrogen bonds were included (cut-off 4 Å). Cuts through covalent bonds were terminated with hydrogen atoms. The **H_{ox}** state has a total spin of 1/2 originating from strong antiferromagnetic coupling of 18 electrons within the cubane (two high-spin Fe^{III} and two high spin Fe^{II}) that in turn couple weakly with one unpaired electron at the $[2Fe]_H$ site (Figure 1b).

Table S2. Residues included in ONIOM calculations. All LYS amino acids are protonated.

Cp	*Dd*
CYS299	GLN71
CYS300	CYS178
GLY302	CYS179
TRP303	GLY181
CYS499	TRP182
CYS355	ALA377
THR356	CYS378
LYS358	CYS234
LYS359	ALA236
PRO324	LYS237
PHE417	PRO203
CYS503	PHE296
LYS322	CYS382
	LYS201
	LYS238

The geometry optimisations were performed with usual set-up. In the ONIOM calculations described in the main body of the manuscript the hydrogen atoms were used as a link atoms between high- and low-layers. The distance scaling factors for C-C and C-N(amide) bonds were 0.709 and 0.729, respectively. The starting orbitals for calculations were converted with an in-home written Perl script from converged Turbomole alpha and beta ASCII files to input files that can be used with asc2mov program (part of official NWChem 6.3 distribution) to

obtain binary orbital files of NWChem. In this way we kept the same broken-symmetry coupling in all calculations.

Intermediate model set-up for Gibbs' free energy corrections calculations and explanation of the role of the [Fe_4S_4] cubane

Because we kept some of the atoms fixed in our model and approximate nature of the transition states the direct usage of the harmonic approximation to account for Gibbs' free energy corrections was not possible. We decided to construct a model (blue and red atoms in Figure 1a) for which the Gibbs free energy corrections can be evaluated easily. The system comprised from entire H-cluster along with anchoring CYS residues that were replaced with CH_3-S^- fragments as usual. The oxygen molecule was optimised separately. We noted that the oxygen molecule already lost some translation entropy due to penetration of the protein and according to MD simulations[17a] this effect is about 4 kcal/mol. In our calculations this can be estimated by noting that O_2 molecule lost some translation entropy due to entering into the binding pocket (average radius of ~3 Å[17]). In standard condition one oxygen molecule occupies $V_0 = 3.73 \cdot 10^{-26}$ m^3 while the protein pocket volume is $V_p = 1.13 \cdot 10^{-28}$ m^3. We thus scaled the translation partition function to account for this reduced volume what resulted in the -*TS* contribution of 10.4 kcal/mol at 298.15 K. Further corrections for zero-point energy change and enthalpy change were small: 1.5 kcal/mol and -1.0 kcal/mol, respectively. Thus, we found that 9.9 kcal/mol need to be added to the electronic binding energy to account for the free energy change. The same shift was applied to the electronic activation energy what constitute an upper limit for $\Delta G^{\ddagger}$.

We observed dramatic change (~10 kcal/mol) between binding energies of the small (Figure 2) and large models (Figure 3). Thus we looked at the binding energies in the intermediate model (one used for ΔG corrections) and found that ΔE goes down to -20.6 kcal/mol in comparison to the small model (to -5.2 kcal/mol). Moreover, the binding energy becomes less favourable by 3 – 5 kcal/mol for the large models in comparison to the medium system. This is mainly due to inclusion of the counter-charges and partially due to some less pronounced screening effects.

Comparison of most important interatomic distances for small, intermediate and large models

Table S3. Most important interatomic distances.

	Small		Medium		Cp			Dd		
					Free		Bound	Free		Bound
	Free	Bound	Free	Bound	Exp.	Calc.		Exp.	Calc.	
$Fe_p...Fe_d$	2.53	2.56	2.50	2.56	2.55	2.51	2.52	2.55	2.53	2.56
$Fe_d...\mu\underline{C}O$	1.83	1.96	1.89	2.06	1.99	1.91	2.06	---	1.92	2.07
$Fe_p...\mu\underline{C}O$	2.20	1.99	2.02	1.90	1.91	1.97	1.89	---	1.97	1.89
$Fe^1...Fe^2$	---	---	2.61	2.61	2.72	2.64	2.60	2.69	2.65	2.63
$Fe^3...Fe^4$	---	---	2.63	2.63	2.68	2.63	2.65	2.68	2.64	2.66
$O^1...O^2$	---	1.29	---	1.31	---	---	1.31	---	---	1.30
$Fe_d...O^1$	---	1.95	---	1.92	---	---	1.89	---	---	1.90
ang(Fe_d-O^1-O^2)	---	126	---	123	---	---	127	---	---	123

Calculations of oxygen inactivation rates

Within the steady-state approximation, the rate for ligand binding is given by $k_{in} = k_{+1}k_2/(k_{-1}+k_2)$ where k_{+1} is the bi-molecular rate constant for ligand diffusion from the solvent to the active site cavity, k_{-1} is the rate constant for the reverse process and k_2 is the rate constant for chemical attachment of the ligand initially located in the active site cavity. Converting the computed free energies into k_2 using transition state theory and adopting values for k_{+1} and k_{-1} from previous MD simulations for [NiFe]-hydrogenase,[22] we obtain values for k_{in} of 3.6 $s^{-1}mM^{-1}$ and 1.2 $s^{-1}mM^{-1}$ for *Cp* and *Dd* enzymes, respectively. We believe that this should provide good first approximation to the corresponding diffusion rates in [FeFe]-hydrogenases because for small k_{+2} k_{in} is proportional to k_{+1}/k_{-1} which in turn only depends on the size of the active site cavity (for the same concentration).

References

[1] a) P. A. M. Dirac, *Proc. Royal Soc. (London)* **1929**, *A123*, 714; b) J. C. Slater, *Phys. Rev.* **1951**, *81*, 385; c) S. H. Vosko, L. Wilk, M. Nusair, *Can. J. Phys.* **1980**, *58*, 1200; d) A. D. Becke, *Phys. Rev.* **1988**, *A38*, 3098; e) J. P. Perdew, *Phys. Rev.* **1986**, *B33*, 8822.

[2] a) A. Hellweg, C. Hättig, S. Höfener, W. Klopper, *Theor. Chem. Acc.* **2007**, *117*, 587; b) F. Weigend, R. Ahlrichs, *Phys. Chem. Chem. .Phys.* **2005**, *7*, 3297; c) F. Weigend, M. Häser, H. Patzelt, R. Ahlrichs, *Chem. Phys. Lett.* **1998**, *294*, 143.

[3] TURBOMOLE V6.5 2011, a development of University of Karlsruhe and Forschungszentrum Karlsruhe GmbH, 1989-2007, TURBOMOLE GmbH, since 2007; available from http://www.turbomole.com.
[4] S. Grimme, J. Antony, S. Ehrlich, H. Krieg, *J. Chem. Phys.* **2010**, *132*, 154104.
[5] S. Grimme, S. Ehrlich, L. Goerigk, J. Comput. Chem. 2011, 32, 1456 – 1465.
[6] a) K. Eichkorn, O. Treutler, H. Ohm, M. Häser and R. Ahlrichs, *Chem. Phys. Lett.* **1995**, *240*, 283; b) M. Sierka, A. Hogekamp and R. Ahlrichs, *J. Chem. Phys.* **2003**, *118*, 9136.
[7] V. S. Bryantsev, W. A. de Jong, K. C. Cossel, M. S. Diallo, W. A. Goddard III, G. S. Groenewold, W. Chien, M. J. Van Stipdonk, *J. Phys. Chem. A* **2008**, *112*, 5777 – 5780.
[8] B. O. Roos, P. R. Taylor, P. E. M. Siegbahn, *Chem. Phys.* **1980**, *48*, 157.
[9] a) C. Lee, W. Yang and R. G. Parr, *Phys. Rev. B* **1988**, *37*, 785; b) A. D. Becke, *J. Chem. Phys.* **1993**, *98*, 5648.
[10] T. Yanai, D. P. Tew, N. C. Handy, *Chem. Phys. Lett.* **2004**, *393*, 51 – 57.
[11] a) J. Heyd, G. E. Scuseria, M. Ernzerhof, *J. Chem. Phys.* **2003**, *118*, 8207 – 8215.
[12] a) H. Iikura, T. Tsuneda, T. Yanai, K. Hirao, *J. Chem. Phys.* **2001**, *115*, 3540 – 3544; b) Y. Tawada, T. Tsuneda, S. Yanagisawa, T. Yanai, K. Hirao, *J. Chem. Phys.* **2004**, *120*, 8425 – 8433; T. Leininger, H. Stoll, H.-J. Werner, A. Savin, *Chem. Phys. Lett.* **1997**, *275*, 151 – 160.
[13] M. A. Rohrdanz, J. M. Herber, *J. Phys. Chem.* **2008**, *129*, 34107 – 34116.
[14] Zhao, Y.; Truhlar, D. G. *J. Phys. Chem. A* **2005**, *109*, 5656-5667.
[15] A. Silakov, B. Wenk, E. Reijerse, W Lubitz, *Phys. Chem. Chem. Phys.* **2009**, *11*, 6592 – 6599.
[16] U. Ryde, L. Greco, L. De Gioia, *J. Am. Chem. Soc.* **2010**, *132*, 4512 – 4513.
[17] a) P. Wang, R. B. Best, J. Blumberger, *Phys. Chem. Chem. Phys.* **2011**, *13*, 7708–7719; b) P. Wang, J. Blumberger, *Proc. Natl. Acad. Sci. U. S. A.* **2012**, *109*, 6399–6404

Cartesian coordinates

Small model bound with O_2
30

C	24.0741253	13.6416776	21.6497086
Fe	25.7722444	13.2774239	21.3817937
C	26.3305053	15.0640910	21.8053315
N	26.6443973	16.1849783	21.9629613
O	22.9517742	13.9116188	21.7870067
S	25.6507918	13.9416274	19.2062642
C	24.9532085	15.6243317	19.0520726
S	25.3215588	11.0447645	20.9595917
C	26.2767409	10.4549411	19.4467818
N	27.7108674	10.4339614	19.5099663
C	28.3403142	11.7183273	19.6457139
S	28.0409523	12.7007069	21.2186985
Fe	26.8559571	11.3624494	22.6970697
C	27.9657967	11.7464547	24.0203372
O	28.6859730	11.9935518	24.8899340
C	25.8460281	12.9128484	23.3357728
O	25.4787072	13.4069021	24.3321878
O	27.7792698	9.6849081	22.3523201
O	28.6014342	9.0924403	23.1496779
C	25.6661628	10.3754630	23.8385656
N	24.9456906	9.7605920	24.5294547
H	28.0137906	9.8135916	20.2667451
H	25.9067759	9.4319035	19.2960668
H	25.9562114	11.0688607	18.5899879
H	29.4308547	11.5913295	19.6217801
H	28.0449174	12.3667013	18.8062417
H	25.6561406	16.2817313	19.5765461
H	23.9759558	15.6825388	19.5425707
H	24.8823226	15.8818154	17.9885155
H	24.5633502	13.2944961	18.7069469

Small model approximate TS
30

C	-1.2408337	2.0735017	1.2014135
Fe	-0.0392051	1.2255647	0.2409911
C	1.4238164	2.1208859	1.1012019
N	2.3011694	2.7543554	1.5588059
O	-2.0270218	2.6760039	1.8113552
S	-0.0007573	2.8573568	-1.3284189
C	0.2681116	4.5036963	-0.5778950
S	-1.6457960	-0.1053863	-0.7633855
C	-1.3332529	-0.2224849	-2.6176597
N	-0.1295114	-0.8544905	-3.0752657
C	1.0958846	-0.2096534	-2.6946599
S	1.5321282	-0.1039159	-0.8701338
Fe	-0.0014523	-1.3137468	0.3526788
C	1.2824467	-2.2005682	1.1780037
O	2.1164220	-2.7944960	1.7178349
C	0.0028620	-0.1045387	1.7977506
O	0.0256216	-0.0206446	2.9675930
O	-0.1006103	-3.1540768	-1.1846298
O	0.4027028	-4.2880599	-0.9474459
C	-1.3776604	-2.2788390	1.2632028
N	-2.2195349	-2.8862603	1.8101942
H	-0.1164608	-1.8386930	-2.7940725
H	-2.1999679	-0.7842798	-2.9905985
H	-1.3850105	0.8022478	-3.0190986
H	1.9419377	-0.7511670	-3.1381383
H	1.1045846	0.8239799	-3.0748096
H	1.2497654	4.4461555	-0.0933307
H	-0.4940341	4.7080932	0.1814133
H	0.2565362	5.2622813	-1.3696755
H	-1.3037223	3.0716757	-1.6589832

Small model Fe…O_2 3.5 Å
30

C	-0.9802284	2.4065944	1.1458928
Fe	0.0200237	1.3336807	0.1849564
C	1.6343679	2.1288189	0.8409426
N	2.6094945	2.6958406	1.1708447
O	-1.6332762	3.1541454	1.7542034
S	0.0740091	2.7643369	-1.5648347
C	0.6072567	4.4360107	-1.0454583
S	-1.8074933	0.0778937	-0.5071481
C	-1.7134711	-0.3102399	-2.3420541
N	-0.6200346	-1.1133455	-2.8145802
C	0.6877422	-0.5392318	-2.6495798
S	1.3384570	-0.2602730	-0.9101845
Fe	-0.1690436	-1.1758143	0.5547657
C	1.1012024	-2.1350930	1.3053824
O	1.9305896	-2.7700574	1.8106709
C	0.0056123	0.1302966	1.8727105
O	0.0601970	0.3137918	3.0327441
O	0.4389188	-3.9745087	-1.4570803
O	1.0401125	-4.9523455	-1.0012314
C	-1.4998149	-2.2972598	1.3275472
N	-2.3162019	-3.0305177	1.7490001
H	-0.6546170	-2.0468071	-2.3976819
H	-2.6589621	-0.8279118	-2.5522354
H	-1.7146932	0.6501460	-2.8812857
H	1.4338065	-1.2020516	-3.1070361
H	0.7241144	0.4361696	-3.1594397
H	1.6144262	4.3050779	-0.6325306
H	-0.0519199	4.8216664	-0.2606018
H	0.6167540	5.0978917	-1.9198266
H	-1.2219837	3.1061312	-1.8039879

Small model catalyst
28

C	24.0020377	13.9633444	21.5099679
Fe	25.6600479	13.4358633	21.3194666
C	26.3179882	15.1594146	21.8275774
N	26.7009857	16.2474963	22.0542535
O	22.9031917	14.3474372	21.5804434
S	25.7099881	14.0142166	19.1656005
C	25.2268437	15.7655985	18.9270720
S	25.0531602	11.2078018	21.1347046
C	26.0044290	10.2720089	19.8034660
N	27.4336683	10.2263452	19.8939038
C	28.1054342	11.4966572	19.8379388
S	27.8800159	12.7044299	21.2613131
Fe	26.5620380	11.6075550	22.8134949
C	26.9169239	9.7442805	23.0617153
N	27.1566304	8.5946042	23.1278706
C	25.4606306	12.9289435	23.4505910
O	24.8497456	13.4289570	24.3205899
C	27.6639528	11.9368128	24.1439653
O	28.3950276	12.1365993	25.0256562
H	27.7083261	9.7033092	20.7325947
H	25.6066928	9.2507816	19.8615609
H	25.7048520	10.7188875	18.8427003
H	29.1907492	11.3298139	19.8086302
H	27.8092713	12.0273563	18.9190878
H	25.9649900	16.3516949	19.4864451
H	24.2339512	15.9497178	19.3510882
H	25.2552439	16.0040513	17.8567029
H	24.5792930	13.4984961	18.6061103

O_2 molecule
2

O	0.0000000	0.0000000	-0.6101978
O	0.0000000	0.0000000	0.6101978

Medium model bound with O_2
52

C	0.3866894	-4.5438154	-2.4291242
S	-0.4418310	-3.6887622	-3.8284618
C	-3.5908060	2.5216095	-1.6053682
S	-3.4390395	1.7058053	-3.2461491
C	3.2184556	0.2214243	-5.9717169
S	2.7012186	1.6875337	-4.9936755
C	2.8699310	1.5725917	0.1529969
S	1.5721869	0.4073911	0.6897660
Fe	0.5473793	1.1375725	2.6439882

Fe	-0.5013052	-0.0274806	4.6725964
S	1.6314554	-0.4852444	3.9321899
S	-1.2049646	-0.3510635	2.4805276
C	-0.5649674	-2.0102568	1.9066011
C	1.5907683	-2.1007164	2.9917523
O	-0.8556806	3.1642139	1.0880458
N	2.9566219	2.9743941	3.2381455
O	-0.4685738	2.9762485	4.6958605
N	-3.3645841	0.8606285	5.4443629
O	0.5146912	0.8046985	7.2786486
C	-0.3044383	2.3477940	1.7002674
C	2.0383932	2.2772577	3.0105381
C	-0.2229339	1.9512219	4.1766277
C	-2.2830836	0.5331407	5.1250599
C	0.1134484	0.4541710	6.2442186
Fe	-0.1241136	-1.5606215	-3.1880583
Fe	-1.4216168	0.7847054	-2.9709134
Fe	0.4477025	0.1327300	-1.2753482
Fe	1.0644144	0.7017143	-3.7979962
S	0.1509811	2.1492814	-2.2784658
S	1.9235357	-1.0507307	-2.5727397
S	-0.6340111	-0.1526050	-4.9094564
S	-1.4469595	-0.9124726	-1.4274070
H	-3.5081909	1.7821900	-0.7989123
H	-2.7984559	3.2679654	-1.4688713
H	1.4483315	-4.2665905	-2.3837678
H	0.2972928	-5.6328946	-2.5676843
H	3.5412124	-0.5871471	-5.3010820
H	4.0565566	0.5014404	-6.6296705
H	2.4213451	2.5384766	-0.1050900
H	3.5873794	1.7050233	0.9694820
H	2.2403022	-2.7658027	3.5779287
H	2.0579004	-1.8950208	2.0160845
H	-0.0785882	-1.8475012	0.9342279
H	-1.4767261	-2.5973402	1.7359879
H	-0.0824860	-4.2686607	-1.4752868
H	3.3359526	1.1384497	-0.7403503
H	-4.5737612	3.0124777	-1.5413289
H	2.3872058	-0.1522285	-6.5856934
N	0.3141204	-2.7287779	2.7924913
H	-0.1495711	-2.8484122	3.6996698
O	-0.9770136	-1.7970545	5.2496703
O	-0.6944451	-2.2665825	6.4395886

Medium model catalyst
50

C	20.5565749	13.5800613	18.9223892
S	19.1352137	14.4931286	18.1992968
C	17.3103724	18.9059563	23.5147001
S	16.5655444	18.8695986	21.8335800
C	20.5839200	19.5185612	15.7374612
S	20.7258943	20.5833375	17.2280734
C	23.7420867	19.1914353	21.5590591
S	22.9156034	17.6553412	22.0965371
Fe	22.9602349	17.4483778	24.3790160
Fe	23.1786489	15.5423097	25.9832007
S	24.6515853	15.8185295	24.2461998
S	21.4513780	15.7081055	24.4928363
C	21.7572637	14.4250516	23.1679825
C	24.1584828	14.5934647	22.9271602
O	20.9346807	19.5275211	24.5850315
N	25.2129363	19.5218288	24.7300190
O	22.5602911	18.0668037	27.2981530
N	22.4683753	12.6475933	26.7751043
O	25.2690411	15.5373875	28.0174428
C	21.7292503	18.6820655	24.5029375
C	24.3525939	18.7299430	24.5998977
C	22.8092190	17.3485284	26.3886981
C	22.7274768	13.7643773	26.5022827
C	24.4312686	15.5544699	27.1967790
Fe	19.5473232	16.4959998	19.1375981
Fe	18.4389544	18.4050905	20.6893673
Fe	20.9597854	17.7647840	20.9395960
Fe	20.0962393	19.0421038	18.7576143
S	20.0768425	19.8593980	20.8999028
S	21.5712073	17.2761294	18.7861470
S	18.1145587	18.1275607	18.4358325
S	19.3277660	16.4231099	21.4232071
H	17.7203442	17.9197328	23.7688254
H	18.1253398	19.6383873	23.5675874
H	21.5054409	14.0536046	18.6369181
H	20.5432368	12.5402434	18.5602447
H	21.2829708	18.6731116	15.8048386
H	20.8224336	20.1133305	14.8409258
H	23.1813857	20.0632498	21.9150239
H	24.7527968	19.2081439	21.9821096
H	25.0556628	13.9728333	22.7878977
H	23.9735354	15.1767918	22.0114167
H	21.6146548	14.9419916	22.2084607
H	20.9456103	13.6984908	23.3000816
H	20.4904160	13.5743196	20.0184480
H	23.7567725	19.1881305	20.4615701
H	16.5317234	19.1730294	24.2453743
H	19.5661768	19.1150018	15.6444260
N	23.0237050	13.7431373	23.1842687
H	23.1369054	13.2714325	24.0892821

Large model *Dd* bound with O_2
288

N	3.0051399	34.4231988	30.6632722
C	3.7913981	33.8932438	29.5537096
C	4.5989974	35.0639804	28.9639838
O	5.7754103	35.0161875	28.6741444
C	2.8858342	33.3821182	28.4303783
S	1.6883169	32.1015177	29.0119096
Fe	0.1879423	31.5874075	27.3002549
Fe	-2.2501146	30.8211679	27.1631453
C	-2.4682405	29.2522807	26.1292367
N	-2.5330506	28.2326300	25.5397959
C	0.5595802	33.2610209	26.4702528
N	0.7850108	34.2982456	25.9726523
C	-1.5644070	32.3016467	30.1561940
S	-1.5970494	32.6847751	28.3244831
C	-0.8832471	29.9790180	30.1480958
S	-0.6181224	29.6951106	28.3082938
C	1.5030054	30.7652561	26.4887576
O	2.3808582	30.2397990	25.9394784
C	-3.3475694	31.8068826	26.1840221
O	-4.0584368	32.4463224	25.5332005
C	-0.8212078	31.2742283	25.7293133
O	-0.8108876	31.2517882	24.5591332
N	-1.8412266	30.9533751	30.5458592
H	-2.7867322	30.6780999	30.2709725
H	2.2591186	34.1839860	28.0182426
H	3.4878732	32.9562085	27.6193951
H	4.5052548	33.0975582	29.8350662
H	3.5231700	34.3849506	31.5398014
H	2.1374879	33.8950425	30.7811463
H	3.9878343	35.9945321	28.8466458
H	-0.5668733	32.6038391	30.5019297
H	-2.3060054	32.9841788	30.5841480
H	0.1081333	30.2211437	30.5579800
H	-1.1897883	28.9893679	30.5071502
N	-6.3038277	27.7295931	25.3998942
C	-6.8607644	28.5279232	24.2803053
C	-6.1109966	28.2269842	23.0159870
O	-6.5993067	27.8336985	21.9723951
C	-6.6688131	30.0069690	24.6847367
C	-6.8556553	29.9490900	26.2044460
C	-6.1788213	28.6272451	26.5899797
N	-3.9563126	37.8036722	29.5783773
C	-3.6905768	36.3592305	29.5654593
C	-2.1989988	36.1609796	29.7529832
O	-1.7098044	35.3974295	30.5606747
C	-4.1604937	35.5841340	28.2933879
C	-5.5402269	35.0156355	28.4808370
C	-6.6832180	35.8297944	28.5302364
C	-5.6892199	33.6412881	28.7025621
C	-7.9358341	35.2817595	28.8163269
C	-6.9400343	33.0874405	28.9752664
C	-8.0682013	33.9083445	29.0423171
H	-1.5476073	36.7757509	29.0793882
H	-4.1731970	35.9163566	30.4476329
H	-3.7666633	38.2094334	28.6588598

H	-4.9388528	37.9817899	29.7887803
H	-4.1132145	36.2710961	27.4331184
H	-3.4594364	34.7616714	28.0795303
H	-4.8188873	32.9859179	28.6493947
H	-7.0197752	32.0120556	29.1375025
H	-9.0473393	33.4809545	29.2635616
H	-8.8132427	35.9290343	28.8610251
H	-6.5933292	36.9015977	28.3402724
H	-6.4024774	30.8030765	26.7216203
H	-7.9283780	29.9263542	26.4503894
H	-5.6424241	30.3273756	24.4441940
H	-7.3660890	30.6827821	24.1722256
H	-5.1196515	28.7901312	26.8259300
H	-6.6597032	28.1600590	27.4610289
H	-5.3610346	27.4177726	25.1457384
H	-7.9286008	28.3006020	24.1268415
H	-5.0025712	28.3887752	23.1120515
N	-9.7991368	23.1394057	27.6647167
C	-8.5535947	23.2433916	26.9124843
C	-7.8199956	24.5669862	27.0459848
O	-8.2135087	25.4681991	27.7562033
C	-8.7808087	23.0008488	25.4202789
C	-9.2320795	21.5771526	25.1348775
C	-9.1776659	21.3003700	23.6389246
C	-9.4623577	19.8487607	23.3205867
N	-9.0872760	19.5893949	21.8791947
H	-6.8903884	24.6854047	26.4339651
H	-9.5900418	23.0517285	28.6615239
H	-10.2961924	24.0330757	27.5851767
H	-7.8549925	22.4642239	27.2739054
H	-9.5241027	23.7235124	25.0446419
H	-7.8428773	23.2101499	24.8785358
H	-8.5613394	20.8738478	25.6572871
H	-10.2393953	21.4127895	25.5436930
H	-8.1675949	21.5564758	23.2732276
H	-9.8822669	21.9525313	23.0965074
H	-8.8551882	19.1626209	23.9253056
H	-10.5204506	19.5799975	23.4302248
H	-8.0695775	19.7617739	21.7284897
H	-9.2913597	18.6101091	21.5820618
H	-9.6105264	20.2277101	21.2414408
N	12.7500611	28.2055250	31.7390117
C	13.2850985	29.2173528	32.6592146
C	14.7909916	29.0289836	32.7999816
O	15.4751610	29.5152949	33.6750610
C	12.5905894	29.3433850	34.0254143
C	11.1290263	29.7955118	33.8999874
C	11.0829938	31.1329826	33.1729814
O	11.7644280	32.0918963	33.5319518
N	10.3064246	31.1746812	32.0441842
N	-4.3218897	25.9445343	31.8402397
C	-4.0560365	26.4259649	30.4800533
C	-2.5479986	26.5309852	30.1979830
O	-2.1264278	26.6538589	29.0374834
C	-4.7072250	27.7904620	30.1710251
S	-4.6051489	28.9911446	31.5475350
N	-1.7797434	26.5418694	31.3036149
C	-0.3375336	26.4622879	31.2781486
C	0.1410000	25.0149860	31.1169826
O	-0.6176298	24.0830687	30.9271050
C	0.2291981	27.1926650	32.5064879
S	2.0285392	27.0138154	32.7751783
N	3.4891343	24.1931200	31.2869490
C	4.3148880	23.6739181	32.3813973
C	3.4819980	23.0579870	33.5139812
O	3.8192884	22.0317183	34.1048398
N	2.3634646	23.7668667	33.8326045
C	1.5143482	23.4229328	34.9586091
C	0.8389996	22.0629876	34.7659804
O	0.5648016	21.3020459	35.6648331
C	0.4328817	24.5256961	35.0773555
C	-0.6785211	24.2447820	36.0359713
C	-2.0187682	24.2802233	35.7140183
C	-0.5987553	23.8711218	37.4263006
N	-2.7768406	23.9573753	36.8254097
C	-1.9374400	23.6918849	37.8901593
C	0.4591135	23.6371338	38.3223781
C	-2.2324343	23.2915514	39.1965800
C	0.1702382	23.2359288	39.6213552
C	-1.1605595	23.0636064	40.0546797
N	3.5743045	28.8643715	35.2413246
C	4.7772556	28.1086014	35.6055552
C	6.0379966	28.9799838	35.4509800
O	7.1202775	28.6649297	35.9525283
C	4.9461437	26.8809781	34.7048668
N	5.8424451	30.0603088	34.6600363
C	6.8897472	30.9955806	34.4066093
C	6.8279962	32.1989820	35.3329802
O	5.9310022	32.4285409	36.1139572
C	6.9139316	31.4752564	32.9484684
S	7.2178056	30.0726548	31.8082889
H	-3.2612758	28.9512275	31.7123621
H	-4.2684531	28.2279745	29.2665926
H	-5.7856858	27.6651579	30.0057535
H	-4.4313162	25.7146438	29.7274302
H	-4.8343371	25.0659775	31.8422990
H	-4.8411046	26.6556269	32.3649073
H	-2.3003889	26.2448225	32.1378727
H	0.0299477	26.9862771	30.3743503
H	1.2481593	24.8791062	31.1682116
H	-0.0286968	28.2565677	32.4328481
H	-0.2511397	26.7995635	33.4157837
H	4.8758986	24.5164075	32.8158958
H	3.9461799	24.9784825	30.8195133
H	3.3018802	23.4677687	30.5952557
H	2.2017064	24.6448990	33.3259756
H	2.1030859	23.3574113	35.8862433
H	0.9591745	25.4635556	35.3259256
H	-0.0024758	24.6665771	34.0746499
H	-2.4939586	24.5065626	34.7637655
H	-3.7863986	23.9141942	36.8516093
H	1.4945869	23.7502632	38.0022575
H	0.9862111	23.0376999	40.3167800
H	-1.3535371	22.7389710	41.0776806
H	-3.2619877	23.1515131	39.5297854
H	0.5781215	21.8415376	33.6963646
H	10.2082935	32.0857083	31.6030312
H	9.5389793	30.5135886	31.8898884
H	10.6996121	29.9343186	34.9031943
H	10.5118885	29.0417254	33.3886016
H	13.1471001	30.0737426	34.6276615
H	12.6394103	28.3756056	34.5498058
H	13.1976826	30.1935268	32.1433799
H	12.5660924	27.3346190	32.2441688
H	11.8535647	28.5187307	31.3647419
H	15.2349883	28.3911271	31.9897631
H	5.9661162	31.9637798	32.6875925
H	7.7217319	32.2071134	32.8074026
H	7.8426968	30.4800592	34.6320319
H	7.6998426	32.8974291	35.2308969
H	4.8672627	30.2227400	34.3886518
H	4.7742009	27.7760339	36.6581778
H	2.8779692	28.2862552	34.7634193
H	3.1470384	29.3213717	36.0451833
H	5.8985151	26.3843797	34.9277778
H	4.1243659	26.1681758	34.8603579
H	4.9503574	27.1867313	33.6482246
H	5.0504447	22.9086087	32.0851284
Fe	3.0384339	28.2359472	31.2178791
Fe	5.2578406	29.6550988	30.8750616
S	5.0793266	27.3883436	30.7393156
S	3.3905382	30.3516200	31.9378776
N	6.7527136	30.2471748	24.4418654
C	6.7129303	29.1137173	25.3643237
C	7.2259960	27.8169844	24.7279862
O	6.8161945	27.4199024	23.6293980
C	5.2682769	28.8894940	25.8077594
S	5.1386471	27.5007082	26.9998638
N	8.1862894	27.1370134	25.4195870
C	8.6301363	25.8365264	24.9599165
C	7.6269958	24.7239862	25.3079858
O	7.5113390	23.7351092	24.5623822
C	10.0618746	25.5299284	25.4566457
C	11.0299665	26.6210069	24.9621278
C	10.5269743	24.1366196	25.0284771
C	12.4289536	26.5441003	25.5743540

N	6.9744829	24.8540272	26.4812728
C	6.0363918	23.8320045	26.9395642
C	4.8379972	23.6419868	25.9929854
O	4.2906330	22.5434760	25.8915687
C	5.5676131	24.1356802	28.3616364
N	4.4650370	24.7371761	25.2770315
C	3.4081277	24.6656245	24.2830017
C	3.7139980	23.6499868	23.1799870
O	2.8087286	23.1931643	22.4769385
C	3.1851254	26.0394565	23.6267746
C	2.5021439	27.0597269	24.5428452
C	1.0433755	26.7089730	24.8835934
C	0.7216847	27.0683203	26.3273165
N	-0.6576553	26.6436228	26.7072876
H	4.8822987	29.8036743	26.2654348
H	4.6532617	28.6528897	24.9293824
H	7.3144683	29.3668632	26.2526695
H	7.7173658	30.5002204	24.2196761
H	6.2983571	29.9787692	23.5649897
H	8.4138253	27.4488726	26.3602351
H	11.0939153	26.5472465	23.8588027
H	10.5968710	27.6103405	25.1690884
H	13.0434337	27.3992349	25.2611778
H	12.3761390	26.5630710	26.6724603
H	12.9627093	25.6284545	25.2844800
H	10.0424629	25.5616729	26.5621040
H	11.5257915	23.9199876	25.4274922
H	9.8444558	23.3474946	25.3617368
H	10.5810381	24.0746833	23.9277350
H	8.6281145	25.8773360	23.8592832
H	6.8602887	25.7971751	26.8758191
H	6.4205921	24.2092080	29.0488101
H	5.0087339	25.0807306	28.4063129
H	4.9137060	23.3252021	28.7031634
H	6.5561918	22.8643350	26.9112398
H	4.8018019	25.6611332	25.5782884
H	2.4758519	24.3020902	24.7439677
H	4.1646756	26.4233015	23.2966637
H	2.5785184	25.8761740	22.7234380
H	3.0801100	27.1485261	25.4768250
H	2.5432624	28.0514982	24.0721779
H	0.8783145	25.6273830	24.7373922
H	0.3489948	27.2251600	24.2040336
H	1.4344982	26.5947057	27.0161516
H	0.7761580	28.1484601	26.5030643
H	-0.8939757	26.8130423	27.7120607
H	-1.4068244	27.2019189	26.1549579
N	5.0242786	23.3741020	22.9557064
C	5.3642429	22.3747236	21.9707643
C	4.9599972	20.9759882	22.4319874
O	5.2319444	19.9755822	21.8004416
C	6.8539715	22.4304230	21.6495952
C	7.2313008	23.7849935	21.0632080
C	8.7388907	23.8959253	20.8584694
C	9.0870844	25.2235056	20.2240793
N	10.5803801	25.2653882	19.9202543
H	-0.7955314	25.6436214	26.5266715
H	5.7292165	23.6138275	23.6592127
H	4.4100505	20.9417822	23.4025989
H	4.7707181	22.5707254	21.0600929
H	7.4287871	22.2598263	22.5730081
H	7.0845089	21.6135512	20.9509290
H	6.7092213	23.9339254	20.1027862
H	6.8972618	24.5864802	21.7391271
H	9.2406960	23.7976742	21.8361517
H	9.0902968	23.0677868	20.2167100
H	8.8731333	26.0740105	20.8844540
H	8.5766915	25.3776866	19.2645033
H	10.8389634	24.5010780	19.2753984
H	10.8754330	26.1605808	19.4977965
H	11.1206084	25.1313765	20.7918890
Fe	3.0070534	30.4014767	29.7825829
Fe	4.3009880	28.2855798	28.9068232
S	5.0565953	30.4234745	28.8495856
S	2.0683953	28.3866613	29.2855446
O	-3.6076275	30.1719087	28.3294623
O	-4.7714361	30.7502404	28.4422042

Large model *Dd* Fe… O_2 3.5 Å
288

N	2.9778871	34.5060855	30.6806709
C	3.7297405	33.9433104	29.5637804
C	4.5989987	35.0639902	28.9639919
O	5.7594295	34.9359537	28.6361650
C	2.7891451	33.4909111	28.4470501
S	1.5828681	32.1953118	28.9865509
Fe	0.1202712	31.7589080	27.2464253
Fe	-2.2999994	31.1529913	26.8379925
C	-2.7098680	29.5913431	25.8867613
N	-2.8525898	28.5605368	25.3293120
C	0.5441097	33.4292538	26.4442935
N	0.7870219	34.4735656	25.9688303
C	-1.7922434	32.3818458	30.0192440
S	-1.6913693	32.8680943	28.2118726
C	-1.1860874	30.0466958	29.9262215
S	-0.8636641	29.8512638	28.0914676
C	1.4067607	30.8867144	26.4451623
O	2.2670101	30.3097136	25.9173099
C	-3.3419632	32.2320162	25.9155486
O	-3.9983475	32.9553070	25.2885085
C	-0.8900930	31.4708067	25.5782299
O	-0.7123068	31.4502324	24.4162427
N	-2.1355988	31.0276254	30.3377534
H	-3.0813028	30.8001561	30.0197738
H	2.1656745	34.3194137	28.0862182
H	3.3585452	33.0924868	27.5994549
H	4.4023249	33.1090851	29.8361722
H	3.4946622	34.4308913	31.5556150
H	2.0836576	34.0243921	30.7977068
H	4.0522690	36.0367897	28.8725577
H	-0.8029811	32.6236439	30.4332065
H	-2.5279224	33.0714685	30.4465217
H	-0.2054660	30.2588801	30.3777616
H	-1.5152124	29.0470205	30.2357982
N	-6.6888102	27.9694363	25.3502183
C	-6.8256502	28.8738534	24.1820889
C	-6.1109983	28.2269921	23.0159935
O	-6.6194690	27.9495854	21.9441385
C	-6.1388829	30.1921131	24.5989446
C	-6.3282439	30.2190049	26.1188063
C	-6.1601864	28.7445467	26.5010410
N	-3.8341568	37.8849850	29.3965520
C	-3.6822542	36.4269182	29.5273927
C	-2.1989994	36.1609898	29.7529916
O	-1.7611089	35.4905562	30.6669837
C	-4.1876419	35.5827969	28.3286908
C	-5.6218361	35.1322699	28.4490081
C	-6.6608762	36.0256294	28.7581548
C	-5.9437202	33.7795415	28.2714922
C	-7.9751725	35.5784830	28.9002619
C	-7.2611071	33.3324217	28.3909618
C	-8.2811600	34.2280233	28.7136438
H	-1.5156629	36.6489955	29.0136670
H	-4.1949650	36.0991647	30.4432485
H	-3.8665637	38.1690257	28.4169882
H	-4.6824431	38.2173853	29.8505061
H	-4.0387305	36.1706235	27.4064898
H	-3.5469961	34.6941609	28.2111798
H	-5.1457546	33.0711446	28.0406491
H	-7.4833515	32.2762583	28.2456303
H	-9.3077886	33.8755758	28.8217086
H	-8.7644681	36.2877425	29.1547466
H	-6.4377450	37.0850512	28.8924918
H	-5.5978171	30.8695510	26.6128172
H	-7.3374249	30.5769017	26.3744424
H	-5.0650438	30.1419649	24.3636864
H	-6.5618892	31.0609922	24.0817495
H	-5.0943834	28.5142814	26.6414463
H	-6.6966927	28.4674085	27.4191538
H	-7.5848410	27.5454831	25.5803597
H	-7.8802695	29.0373044	23.9047717
H	-5.0414954	27.9840181	23.2438935
N	-9.8376861	23.1617032	27.5424051
C	-8.4877439	23.2075931	26.9942496
C	-7.8199978	24.5669931	27.0459924

O	-8.4219441	25.5879026	27.3132091
C	-8.4889784	22.8621986	25.4970236
C	-8.9742531	21.4508243	25.2170768
C	-8.9616779	21.2093720	23.7142073
C	-9.3698501	19.7994722	23.3485049
N	-9.0872786	19.5894004	21.8792008
H	-6.7416340	24.6004110	26.7500844
H	-9.7973659	23.0538307	28.5573615
H	-10.2749916	24.0766991	27.3847772
H	-7.8462352	22.4786882	27.5194687
H	-9.1386363	23.5886160	24.9800764
H	-7.4758215	23.0014100	25.0834623
H	-8.3161920	20.7215266	25.7181529
H	-9.9817062	21.3231338	25.6402795
H	-7.9446031	21.4027362	23.3296146
H	-9.6272883	21.9305586	23.2106841
H	-8.7911936	19.0392103	23.8887462
H	-10.4399189	19.6086116	23.4996311
H	-8.0657461	19.6909611	21.6830179
H	-9.3816957	18.6433895	21.5463860
H	-9.5919005	20.2978972	21.3002910
N	12.7741652	28.1580843	31.7690060
C	13.2821658	29.1953095	32.6783168
C	14.7909958	29.0289918	32.7999908
O	15.4457963	29.2415671	33.7984556
C	12.5827595	29.3313254	34.0355439
C	11.1249438	29.7920465	33.8977629
C	11.0829969	31.1329913	33.1729907
O	11.7919939	32.0794411	33.5116001
N	10.2689020	31.1914616	32.0728547
N	-4.2012502	25.6404127	31.8331972
C	-4.0338917	26.2858046	30.5284608
C	-2.5479993	26.5309926	30.1979915
O	-2.1891344	26.7782875	29.0375172
C	-4.7857352	27.6253212	30.4266636
S	-4.6123751	28.7062268	31.8903543
N	-1.7609175	26.5214397	31.2823346
C	-0.3213299	26.4692214	31.2398906
C	0.1410000	25.0149930	31.1169913
O	-0.6331742	24.0855846	30.9797881
C	0.2469656	27.2181080	32.4555203
S	2.0485769	27.0317852	32.6931725
N	3.4636009	24.2020190	31.2830829
C	4.2972164	23.6398388	32.3498983
C	3.4819990	23.0579935	33.5139906
O	3.8378042	22.0539556	34.1318331
N	2.3610329	23.7640015	33.8265727
C	1.5203395	23.4222900	34.9594151
C	0.8389998	22.0629938	34.7659902
O	0.5467435	21.3111888	35.6666720
C	0.4552658	24.5381393	35.1016775
C	-0.6643170	24.2582300	36.0515969
C	-2.0016070	24.3275307	35.7229810
C	-0.6008560	23.8625255	37.4370287
N	-2.7732071	24.0041764	36.8248631
C	-1.9459743	23.7071377	37.8908658
C	0.4457073	23.5917126	38.3361808
C	-2.2585079	23.2982358	39.1905094
C	0.1393705	23.1818537	39.6285563
C	-1.1976487	23.0361115	40.0523831
N	3.5789991	28.8169572	35.1737791
C	4.7884050	28.0822556	35.5613293
C	6.0379983	28.9799919	35.4509900
O	7.1140827	28.6831533	35.9756522
C	5.0106723	26.8707399	34.6492715
N	5.8384164	30.0564362	34.6532354
C	6.8737694	31.0034380	34.3909647
C	6.8279981	32.1989910	35.3329901
O	5.9061141	32.4629920	36.0726641
C	6.8366775	31.5294424	32.9476849
S	7.1259759	30.1913635	31.7300944
H	-3.2828453	28.9427210	31.7815430
H	-4.4771402	28.1627035	29.5220451
H	-5.8671685	27.4446420	30.3558118
H	-4.4029970	25.6462759	29.7103103
H	-4.5734390	24.6971953	31.7597359
H	-4.7809719	26.2126617	32.4510331
H	-2.2516532	26.1172473	32.0913943
H	0.0328804	26.9810126	30.3248900
H	1.2462178	24.8644994	31.1516833
H	-0.0149706	28.2798854	32.3667509
H	-0.2282910	26.8354210	33.3720001
H	4.9195678	24.4463601	32.7686674
H	3.8971871	25.0245146	30.8597950
H	3.2892708	23.5144621	30.5512391
H	2.1811845	24.6217238	33.2939147
H	2.1218361	23.3452591	35.8779019
H	0.9968058	25.4611745	35.3717211
H	0.0231988	24.7102218	34.1024866
H	-2.4655382	24.5793448	34.7735507
H	-3.7836342	23.9913110	36.8479559
H	1.4856296	23.6828220	38.0233935
H	0.9459728	22.9557780	40.3264566
H	-1.4045597	22.7051653	41.0706870
H	-3.2931939	23.1780250	39.5155648
H	0.5981673	21.8318564	33.6937790
H	10.1732774	32.1009064	31.6286231
H	9.5020282	30.5307998	31.9218813
H	10.6850989	29.9288739	34.8969175
H	10.5090227	29.0414285	33.3797639
H	13.1372083	30.0598671	34.6417945
H	12.6247391	28.3677408	34.5672346
H	13.1821161	30.1582173	32.1380028
H	12.6576322	27.2781018	32.2780699
H	11.8512551	28.4206196	31.4210945
H	15.2680553	28.6830876	31.8454022
H	5.8709313	32.0087345	32.7420548
H	7.6251573	32.2822647	32.8059668
H	7.8389262	30.4952362	34.5703442
H	7.7326324	32.8597611	35.2746005
H	4.8625168	30.2034149	34.3739615
H	4.7579116	27.7371553	36.6087354
H	2.9390476	28.2512255	34.6085772
H	3.0786113	29.1926131	35.9770967
H	5.9601026	26.3825266	34.9015680
H	4.1927059	26.1451816	34.7611394
H	5.0519602	27.1933462	33.5978928
H	4.9810648	22.8401631	32.0237334
Fe	2.9646460	28.3404851	31.1530834
Fe	5.1780596	29.7346096	30.7846196
S	4.9840137	27.4721868	30.6664603
S	3.3390670	30.4516639	31.8768053
N	6.6809067	30.2253085	24.4133479
C	6.6532329	29.0986675	25.3445470
C	7.2259980	27.8169922	24.7279931
O	6.8551434	27.4174572	23.6160669
C	5.2002409	28.8279527	25.7427969
S	5.0871631	27.4909213	26.9923848
N	8.1779339	27.1423805	25.4393791
C	8.6333210	25.8416491	24.9806281
C	7.6269979	24.7239931	25.3079929
O	7.5165014	23.7367536	24.5609629
C	10.0565466	25.5256911	25.4968050
C	11.0346915	26.6097518	25.0075979
C	10.5124627	24.1256840	25.0789223
C	12.4646719	26.4588612	25.5274258
N	6.9682371	24.8568230	26.4784243
C	6.0311799	23.8382303	26.9409241
C	4.8379986	23.6419934	25.9929927
O	4.3069705	22.5340729	25.8895170
C	5.5552515	24.1508814	28.3591384
N	4.4409347	24.7280645	25.2850361
C	3.3621342	24.6084429	24.3229589
C	3.7139990	23.6499934	23.1799935
O	2.8219676	23.2039431	22.4517697
C	2.9797739	25.9892430	23.7705162
C	2.2445882	26.8687979	24.7882763
C	0.8082623	26.4205563	25.0666012
C	0.2417815	27.1719090	26.2595409
N	-1.1813973	26.8047791	26.5273073
H	4.7585996	29.7437813	26.1438486
H	4.6291193	28.5258586	24.8533551
H	7.2155652	29.3784907	26.2500322
H	7.6421913	30.5093415	24.2154694
H	6.2618758	29.9317086	23.5268866
H	8.3789090	27.4472374	26.3885512

H	11.0330883	26.5960005	23.9001238
H	10.6468054	27.5985372	25.2915927
H	13.0812276	27.3181836	25.2302190
H	12.4825813	26.4063609	26.6257339
H	12.9535306	25.5524197	25.1442949
H	10.0264464	25.5591128	26.6019216
H	11.5015464	23.8982043	25.4952269
H	9.8158984	23.3461488	25.4055983
H	10.5824930	24.0563555	23.9795913
H	8.6502934	25.8893080	23.8809046
H	6.8503831	25.8011210	26.8674523
H	6.4040890	24.2239571	29.0515056
H	4.9986850	25.0976113	28.3956513
H	4.8963453	23.3438728	28.6996446
H	6.5511585	22.8704845	26.9217791
H	4.7480411	25.6617351	25.5912280
H	2.4896463	24.1363756	24.8026339
H	3.9007286	26.4921558	23.4318674
H	2.3517277	25.8310817	22.8816943
H	2.8080737	26.8950989	25.7353357
H	2.2261742	27.9078512	24.4288690
H	0.7817813	25.3359244	25.2755923
H	0.1798638	26.5902407	24.1771694
H	0.8244430	26.9693941	27.1679285
H	0.2613764	28.2567510	26.1057681
H	-1.4426755	26.9691357	27.5265569
H	-1.8668095	27.4076376	25.9415549
N	5.0266975	23.3766758	22.9688093
C	5.3706810	22.3777516	21.9823334
C	4.9599986	20.9759941	22.4319937
O	5.2212708	19.9810046	21.7877078
C	6.8617168	22.4247974	21.6665990
C	7.2424493	23.7734460	21.0714295
C	8.7465681	23.8745222	20.8400245
C	9.0829687	25.1981892	20.1923664
N	10.5803831	25.2653953	19.9202599
H	-1.3472057	25.8119936	26.3337382
H	5.7267282	23.6116973	23.6783261
H	4.4172972	20.9344600	23.4065700
H	4.7838403	22.5793002	21.0688795
H	7.4351485	22.2567143	22.5911837
H	7.0881189	21.6028886	20.9722925
H	6.7049851	23.9239069	20.1198092
H	6.9263457	24.5792441	21.7508092
H	9.2653419	23.7805576	21.8092869
H	9.0853767	23.0426572	20.1964102
H	8.8385103	26.0535911	20.8356322
H	8.5895331	25.3275772	19.2204018
H	10.8721853	24.4833104	19.3115154
H	10.8636428	26.1508378	19.4696853
H	11.1033610	25.1784881	20.8082887
Fe	2.9109440	30.5041229	29.7258674
Fe	4.1847814	28.3528193	28.8349289
S	4.9463256	30.4833676	28.7548893
S	1.9494118	28.5019857	29.2351798
O	-4.9359356	30.3416119	28.9928699
O	-6.0805277	30.6200945	29.3824414

Large model *Dd* Fe… O_2 2.4 Å

288

N	2.9775739	34.4536436	30.6404552
C	3.7650933	33.9042044	29.5408943
C	4.5989987	35.0639902	28.9639919
O	5.7851707	35.0072247	28.7185796
C	2.8593543	33.3889239	28.4182916
S	1.6600155	32.1126271	29.0181315
Fe	0.1330244	31.5655307	27.3804004
Fe	-2.2500994	30.8211913	27.1630924
C	-2.6409990	29.2405851	26.2233256
N	-2.8349744	28.2222799	25.6592150
C	0.4465188	33.2248167	26.5024041
N	0.6413611	34.2664341	25.9991150
C	-1.6228474	32.2875444	30.2211647
S	-1.6647350	32.6514898	28.3756534
C	-0.9027827	29.9643578	30.2278340
S	-0.6885141	29.6767856	28.3713680
C	1.4122610	30.7286448	26.5270592
O	2.2772751	30.1978654	25.9601051
C	-3.4533924	31.7732961	26.2907282
O	-4.2728571	32.3788594	25.7428590
C	-0.9656998	31.1698987	25.7808823
O	-0.8468349	31.1215093	24.6133719
N	-1.8313399	30.9461468	30.6509032
H	-2.7891854	30.6243828	30.5201732
H	2.2350712	34.1889097	27.9979964
H	3.4586854	32.9494888	27.6122414
H	4.4674654	33.1033414	29.8359669
H	3.4822662	34.4055089	31.5240485
H	2.0942126	33.9499731	30.7473685
H	3.9986132	35.9971920	28.8163294
H	-0.6305875	32.6425392	30.5322902
H	-2.3820701	32.9500095	30.6528461
H	0.1101847	30.2013682	30.5864249
H	-1.2184622	28.9885532	30.6134528
N	-6.1042757	27.7005855	25.4151521
C	-6.7805641	28.4557088	24.3400247
C	-6.1109983	28.2269921	23.0159935
O	-6.6683360	27.8716748	21.9938346
C	-6.6917901	29.9415976	24.7574414
C	-6.8896762	29.8439531	26.2746306
C	-6.1944977	28.5221142	26.6593113
N	-4.0332406	37.7352388	29.6445714
C	-3.7018874	36.3052380	29.5859603
C	-2.1989994	36.1609898	29.7529916
O	-1.6756619	35.4094208	30.5500952
C	-4.1598977	35.5519351	28.2975292
C	-5.5575837	35.0185517	28.4523588
C	-6.6883008	35.8512538	28.4251050
C	-5.7410820	33.6569505	28.7242935
C	-7.9604602	35.3352785	28.6860410
C	-7.0104068	33.1350827	28.9689513
C	-8.1262893	33.9755979	28.9633863
H	-1.5766879	36.7995497	29.0740158
H	-4.1539765	35.8161787	30.4603810
H	-3.8655537	38.1774531	28.7371712
H	-5.0242153	37.8565380	29.8579280
H	-4.0766681	36.2433408	27.4439622
H	-3.4720515	34.7149443	28.0958498
H	-4.8780891	32.9895051	28.7281713
H	-7.1206007	32.0676839	29.1639702
H	-9.1213205	33.5757712	29.1642339
H	-8.8256076	35.9996149	28.6683019
H	-6.5741628	36.9107399	28.1890215
H	-6.4680569	30.7030864	26.8081031
H	-7.9659560	29.7972552	26.5014348
H	-5.6893071	30.3300646	24.5208243
H	-7.4278123	30.5831774	24.2544098
H	-5.1776228	28.6995667	27.0350296
H	-6.7691582	27.9805912	27.4259069
H	-5.1050095	27.6129931	25.1861092
H	-7.8321596	28.1375851	24.2646724
H	-5.0029094	28.4139117	23.0341352
N	-9.7597425	23.0987114	27.6884408
C	-8.5303473	23.2308080	26.9141979
C	-7.8199978	24.5669931	27.0459924
O	-8.2225926	25.4543780	27.7698118
C	-8.7827381	22.9996191	25.4238121
C	-9.2277429	21.5744070	25.1356491
C	-9.1901942	21.3040464	23.6377418
C	-9.4585163	19.8486490	23.3214801
N	-9.0872974	19.5893945	21.8791939
H	-6.9067097	24.7158152	26.4166110
H	-9.5305611	23.0077456	28.6805740
H	-10.2723551	23.9849176	27.6255543
H	-7.8120986	22.4599047	27.2537841
H	-9.5387480	23.7191169	25.0676897
H	-7.8563876	23.2215161	24.8673896
H	-8.5453859	20.8733704	25.6460391
H	-10.2281229	21.4000083	25.5570853
H	-8.1883263	21.5743420	23.2598832
H	-9.9099520	21.9483427	23.1061910
H	-8.8395190	19.1719670	23.9248267
H	-10.5123627	19.5662332	23.4372893
H	-8.0772176	19.7951874	21.7183192
H	-9.2598115	18.6003383	21.5932655

H	-9.6377255	20.2031405	21.2394668
N	12.7584442	28.2293800	31.7333667
C	13.2864491	29.2257266	32.6757945
C	14.7909958	29.0289918	32.7999908
O	15.4478138	29.2289115	33.7999680
C	12.5829240	29.3359503	34.0338810
C	11.1236267	29.7935023	33.8984004
C	11.0829969	31.1329913	33.1729907
O	11.7801488	32.0847021	33.5219569
N	10.2880016	31.1845489	32.0582687
N	-4.2073621	25.7355058	31.9081998
C	-4.0333708	26.2493057	30.5348308
C	-2.5479993	26.5309926	30.1979915
O	-2.2050266	26.7207885	29.0240557
C	-4.8700222	27.5009341	30.2745416
S	-4.5618646	28.7920396	31.5515166
N	-1.7527149	26.5510140	31.2771238
C	-0.3142691	26.4734452	31.2496695
C	0.1410000	25.0149930	31.1169913
O	-0.6332656	24.0879250	30.9682631
C	0.2523081	27.1917460	32.4861291
S	2.0499343	26.9984917	32.7508190
N	3.4847836	24.1828658	31.2763492
C	4.3103723	23.6608552	32.3704517
C	3.4819990	23.0579935	33.5139906
O	3.8289889	22.0449230	34.1219651
N	2.3584406	23.7624239	33.8225266
C	1.5120180	23.4248683	34.9519980
C	0.8389998	22.0629938	34.7659902
O	0.5453698	21.3164423	35.6708611
C	0.4314496	24.5291169	35.0719578
C	-0.6772842	24.2494085	36.0335057
C	-2.0173486	24.2565426	35.7075272
C	-0.5950858	23.9015907	37.4306315
N	-2.7724598	23.9373356	36.8220632
C	-1.9318676	23.7072547	37.8935612
C	0.4637956	23.7059917	38.3341629
C	-2.2239759	23.3278993	39.2073923
C	0.1778045	23.3276838	39.6410746
C	-1.1509724	23.1392011	40.0735187
N	3.5709995	28.8373904	35.2257997
C	4.7817349	28.0979040	35.6015832
C	6.0379983	28.9799919	35.4509900
O	7.1242729	28.6664750	35.9449940
C	4.9727485	26.8625431	34.7149678
N	5.8323560	30.0633803	34.6679436
C	6.8795417	30.9961418	34.4073707
C	6.8279981	32.1989910	35.3329901
O	5.9277421	32.4432911	36.1057840
C	6.8967784	31.4766514	32.9493985
S	7.1997903	30.0743732	31.8082386
H	-5.1279870	29.7713359	30.7921223
H	-4.5983450	27.9162260	29.2970275
H	-5.9351392	27.2396679	30.2902418
H	-4.3261433	25.4934810	29.7867027
H	-4.3864466	24.7304495	31.9183797
H	-4.9800960	26.2102974	32.3804865
H	-2.2546264	26.2559555	32.1240912
H	0.0624428	26.9919045	30.3466900
H	1.2471686	24.8644754	31.1505689
H	-0.0029012	28.2559361	32.4258488
H	-0.2326876	26.7949765	33.3916015
H	4.8849775	24.4974092	32.7986252
H	3.9227806	24.9939855	30.8355878
H	3.3307776	23.4705505	30.5633315
H	2.1890469	24.6304643	33.3017932
H	2.1033549	23.3628380	35.8783030
H	0.9594114	25.4663874	35.3188535
H	-0.0068592	24.6698315	34.0706268
H	-2.4926133	24.4646151	34.7534903
H	-3.7818997	23.8858692	36.8489684
H	1.4982394	23.8326622	38.0150932
H	0.9946374	23.1616277	40.3437979
H	-1.3414874	22.8332778	41.1028563
H	-3.2516972	23.1750318	39.5397924
H	0.6009740	21.8244481	33.6946679
H	10.1904531	32.0955722	31.6175514
H	9.5210645	30.5224650	31.9058575

H	10.6853515	29.9309017	34.8980565
H	10.5079789	29.0426371	33.3807830
H	13.1332258	30.0569857	34.6526305
H	12.6270454	28.3644368	34.5507593
H	13.2085613	30.2072165	32.1660784
H	12.5851552	27.3469748	32.2214289
H	11.8599299	28.5419689	31.3638440
H	15.2643530	28.6766460	31.8407841
H	5.9472802	31.9641555	32.6931234
H	7.7029948	32.2097347	32.8058047
H	7.8321354	30.4772933	34.6266422
H	7.7112320	32.8839804	35.2379719
H	4.8528958	30.2269556	34.4115830
H	4.7693239	27.7758998	36.6568491
H	2.9188710	28.2682691	34.6782243
H	3.0863798	29.2237706	36.0337971
H	5.9243966	26.3734343	34.9568762
H	4.1521975	26.1469472	34.8645211
H	4.9917971	27.1577621	33.6549828
H	5.0350637	22.8867368	32.0715882
Fe	3.0419002	28.2388272	31.1973366
Fe	5.2449088	29.6671153	30.8600078
S	5.0829292	27.3989358	30.7108295
S	3.3798579	30.3500073	31.9335905
N	6.7422729	30.2362086	24.4074960
C	6.6967270	29.1158462	25.3467692
C	7.2259980	27.8169922	24.7279931
O	6.8251051	27.4125233	23.6285108
C	5.2472498	28.8851262	25.7770814
S	5.1277431	27.5074855	26.9841387
N	8.1848269	27.1408142	25.4261294
C	8.6332664	25.8388467	24.9702549
C	7.6269979	24.7239931	25.3079929
O	7.5126535	23.7374062	24.5599367
C	10.0602046	25.5309398	25.4811715
C	11.0362076	26.6092854	24.9736914
C	10.5191452	24.1283365	25.0761978
C	12.4350749	26.5385524	25.5871076
N	6.9728533	24.8544396	26.4803354
C	6.0346610	23.8342547	26.9394213
C	4.8379986	23.6419934	25.9929927
O	4.3004799	22.5380038	25.8893305
C	5.5660376	24.1406211	28.3607818
N	4.4484670	24.7337434	25.2842507
C	3.3804580	24.6381758	24.3047454
C	3.7139990	23.6499934	23.1799935
O	2.8159479	23.2002560	22.4622370
C	3.0723005	26.0195269	23.7010307
C	2.3696624	26.9767936	24.6711812
C	0.9172368	26.5951665	24.9939451
C	0.5423357	27.0739078	26.3877268
N	-0.8407291	26.6719067	26.7635941
H	4.8476371	29.8008092	26.2209853
H	4.6414012	28.6305351	24.8965397
H	7.2852794	29.3843471	26.2390347
H	7.7088046	30.5070433	24.2160635
H	6.3279019	29.9395067	23.5196806
H	8.4088504	27.4549295	26.3672015
H	11.1020769	26.5164906	23.8719335
H	10.6070951	27.6039281	25.1638098
H	13.0616122	27.3674849	25.2298615
H	12.3874452	26.6123277	26.6830940
H	12.9535300	25.6016484	25.3399308
H	10.0342583	25.5784554	26.5859740
H	11.5167051	23.9136485	25.4792985
H	9.8338305	23.3477382	25.4237066
H	10.5723416	24.0465062	23.9769886
H	8.6429774	25.8795236	23.8697576
H	6.8596349	25.7978916	26.8735060
H	6.4152790	24.1743910	29.0557425
H	5.0420260	25.1053462	28.4113026
H	4.8793589	23.3515589	28.6878797
H	6.5529985	22.8657542	26.9133504
H	4.7750430	25.6626472	25.5829745
H	2.4773222	24.2164740	24.7753161
H	4.0226904	26.4641983	23.3615101
H	2.4508707	25.8584042	22.8077099
H	2.9458578	27.0311211	25.6094260

H	2.3895038	27.9944442	24.2570865
H	0.7921800	25.4986229	24.9426467
H	0.2281353	27.0262226	24.2507077
H	1.2310312	26.6652549	27.1399602
H	0.5876568	28.1655446	26.4765404
H	-1.0763731	26.8637206	27.7704809
H	-1.5917893	27.2129993	26.2184933
N	5.0249455	23.3743745	22.9639975
C	5.3640230	22.3776963	21.9746492
C	4.9599986	20.9759941	22.4319937
O	5.2395368	19.9767901	21.8020842
C	6.8518047	22.4349294	21.6459594
C	7.2295853	23.7947735	21.0714551
C	8.7365065	23.9014979	20.8594537
C	9.0923750	25.2402497	20.2525219
N	10.5803970	25.2653929	19.9202944
H	-0.9904660	25.6693691	26.6099141
H	5.7281266	23.6089490	23.6709137
H	4.4022704	20.9400539	23.3980402
H	4.7657226	22.5770198	21.0678795
H	7.4321198	22.2529946	22.5637489
H	7.0765519	21.6247499	20.9375913
H	6.7031491	23.9558602	20.1153246
H	6.9018245	24.5900200	21.7576404
H	9.2445816	23.7763534	21.8307822
H	9.0774422	23.0860694	20.1957751
H	8.9043968	26.0774269	20.9374557
H	8.5664343	25.4261981	19.3070126
H	10.8166801	24.4933492	19.2758271
H	10.8763953	26.1544587	19.4854815
H	11.1367481	25.1311568	20.7817456
Fe	2.9859681	30.4131316	29.7794283
Fe	4.2937258	28.3019131	28.8855991
S	5.0339924	30.4438128	28.8373386
S	2.0631996	28.3979480	29.2688942
O	-3.9613113	30.0027089	28.6334110
O	-5.0038314	30.7057219	28.8982854

Large model *Cp* bound with O_2
255

N	21.4425301	10.3371652	28.7861614
C	21.8610833	11.3002625	29.8408761
C	20.6319884	12.0059932	30.3499830
O	20.3181734	12.1274685	31.5206135
C	22.8083848	12.3167055	29.1546158
C	23.3528852	11.5352426	27.9591442
C	22.1235987	10.7331968	27.5265078
N	29.9187011	15.8125760	24.7836297
C	28.5944936	15.3603518	24.3305231
C	27.9879904	16.4944226	23.5202346
O	27.4980566	16.3647637	22.4165750
C	27.5998521	14.9348581	25.4488781
C	27.6434884	13.4598198	25.7524083
C	28.8223200	12.8167895	26.1652117
C	26.4889902	12.6853342	25.5801274
C	28.8460022	11.4380885	26.3817458
C	26.5029476	11.3088616	25.8121278
C	27.6847936	10.6787124	26.2077501
H	24.1743178	10.8683533	28.2620024
H	23.7209717	12.1856203	27.1626269
H	23.5747918	12.6974970	29.8394917
H	22.2271625	13.1788683	28.7897176
H	22.3560090	9.8430266	26.9275282
H	21.4590591	11.3754450	26.9332988
H	22.3532174	10.7907939	30.6826517
H	19.9911622	12.4182489	29.5267506
H	21.7351278	9.3998340	29.0572518
H	25.5663535	13.1681097	25.2566552
H	25.5813591	10.7415435	25.6799345
H	27.7036666	9.6020948	26.3828174
H	29.7743035	10.9546882	26.6906120
H	29.7321432	13.4000816	26.3193469
H	28.7350656	14.5223313	23.6332296
H	30.5617132	15.0280130	24.8770040
H	29.8563610	16.2696522	25.6952224
H	28.0275000	17.4977871	24.0179755
H	27.8222623	15.5327937	26.3493294
H	26.5726368	15.2050677	25.1541506
N	19.0161328	4.5992428	27.9819680
C	18.4916750	5.9627540	27.9695355
C	19.5609890	7.0299960	27.7839844
O	20.7488633	6.7765694	27.7956202
C	17.7628776	6.2823613	29.2929088
C	16.5194916	5.4170737	29.4624349
C	15.8315974	5.6471358	30.8104968
C	14.5065975	4.9079802	30.8705751
N	13.7949922	5.1749970	32.1859820
H	14.3655176	4.8329954	32.9949062
H	12.8624237	4.6989297	32.2294948
H	13.6357015	6.2003099	32.3192572
H	13.8178349	5.2362032	30.0813629
H	14.6299719	3.8195164	30.8020593
H	16.4888042	5.3123698	31.6299042
H	15.6579687	6.7258171	30.9624545
H	16.8136023	4.3615511	29.3547274
H	15.8107267	5.6392162	28.6466972
H	18.4673750	6.0996759	30.1208220
H	17.4949175	7.3511908	29.3281125
H	17.7625500	6.0805007	27.1477583
H	20.0093093	4.6512364	28.2364946
H	18.9962485	4.2044336	27.0407762
H	19.2037161	8.0847476	27.6901287
N	24.1106733	19.2639200	19.6808708
C	23.2246425	20.0439919	20.5408282
C	23.7999866	21.4469880	20.7309884
O	23.1895751	22.3774865	21.2118125
C	22.9494206	19.4906767	21.9489983
S	22.3600986	17.7646002	21.8836871
Fe	22.0688459	16.9312804	23.9966546
Fe	22.5883214	14.7091834	25.0743943
S	24.1846322	15.9532976	23.9959178
S	21.2944479	14.9242171	23.1856411
C	22.3026439	14.3800889	21.6856252
C	24.5037178	15.2016441	22.3040545
O	19.4820902	18.2430604	24.1714137
N	23.4243913	19.3871203	25.2640913
O	21.2694553	16.7759932	26.8180446
N	19.9606765	13.5531588	26.1472597
O	24.1308894	14.9376372	27.5518976
C	20.5006304	17.6960790	24.0725681
C	22.9092818	18.4515744	24.7809231
C	21.7283275	16.4431604	25.7931788
C	21.0181331	13.9324322	25.7932881
C	23.5153499	14.8144691	26.5789505
N	23.6833492	14.1069174	21.8922807
H	23.7931672	13.3041897	22.5137220
H	24.4052990	16.0368649	21.5971433
H	25.5546335	14.8951775	22.3469611
H	21.7983423	13.4578545	21.3728083
H	22.1664589	15.1624676	20.9250212
H	23.8619239	19.4669521	22.5611884
H	22.2155894	20.1136646	22.4735681
H	24.8938526	18.8771356	20.2148912
H	23.6028883	18.4702679	19.2850829
H	22.2588116	20.1626991	20.0204773
H	24.8600378	21.5539213	20.3789050
N	20.5113007	8.9520217	21.4174387
C	20.3538807	9.8016731	22.6122084
C	19.7739888	11.1869938	22.2599874
O	19.4042213	11.9602523	23.1534364
C	21.6378208	9.9770684	23.4147539
S	23.0055522	10.5131677	22.3048419
N	19.6970164	11.4193545	20.9375637
C	19.0137111	12.5421843	20.3364597
C	17.5669902	12.1609932	19.9869888
O	17.2291596	11.0388526	19.6711078
C	19.7555883	12.9885901	19.0685678
S	18.8182471	14.2064077	18.0630372
N	14.5470534	14.0877742	18.1540898
C	15.2790362	14.3489342	16.8980172
C	15.4209914	13.0529926	16.0979910
O	14.4422717	12.5117074	15.5756089
N	16.6642843	12.4961206	16.0946854
C	16.9856593	11.2185829	15.4666383
C	16.0659910	10.0749944	15.9179910

O	15.6810066	9.1910897	15.1876603
C	18.4490474	10.8774263	15.8646907
C	18.8429209	9.4446022	15.6959607
C	19.2714639	8.6285489	16.7213392
C	18.8177339	8.6319042	14.5057583
N	19.5237423	7.3580854	16.2350216
C	19.2477872	7.3231402	14.8817644
C	18.4397947	8.8584736	13.1709395
C	19.3122891	6.2640931	13.9716608
C	18.4989993	7.8068021	12.2635374
C	18.9310122	6.5235857	12.6581368
N	20.7290569	17.7640172	14.8793839
C	21.0485170	19.1636811	14.7117294
C	22.4139874	19.3509892	14.0919920
O	23.2422093	18.4716414	13.9669549
C	21.0464210	20.0297929	16.0009825
S	19.3800677	20.1192371	16.7547729
H	23.7262465	10.9807315	23.3627219
H	21.9128426	9.0306535	23.9003767
H	21.5077695	10.7526819	24.1785791
H	19.6001834	9.3538079	23.2797506
H	20.0907039	8.0350987	21.5556510
H	21.5029009	8.8132257	21.2074442
H	19.8933010	10.5806793	20.3790017
H	18.9958527	13.3776280	21.0541640
H	20.7374247	13.3942695	19.3397666
H	19.9195008	12.1116548	18.4232089
H	16.8353609	13.0017299	20.0428085
H	19.8491699	6.5760199	16.7864271
H	19.6368206	5.2687333	14.2793233
H	18.9589740	5.7177885	11.9241348
H	18.1935127	7.9698977	11.2296973
H	18.0857942	9.8391503	12.8531971
H	19.4015630	8.8586595	17.7747600
H	19.1142603	11.5530987	15.3028205
H	18.5635584	11.1338560	16.9294952
H	16.8869402	11.2869955	14.3720164
H	15.8136619	10.0994266	17.0101797
H	17.4140217	12.9937678	16.5896667
H	14.6978370	15.0602041	16.2964962
H	16.2464057	14.8054888	17.1451441
H	15.1090133	13.4628696	18.7397740
H	13.6972474	13.5619739	17.9220264
H	21.7642606	19.6308398	16.7286330
H	21.3309936	21.0666054	15.7630522
H	20.3252638	19.6394566	14.0186392
H	22.6368448	20.4041436	13.7795548
H	21.5773313	17.2427048	15.1121776
H	20.0515026	17.6419376	15.6344850
Fe	19.1271553	16.1712037	19.0501749
Fe	19.3119532	18.7531676	18.4712058
S	21.0154997	17.2690376	18.4126227
S	17.4085419	17.5202531	18.4987250
N	17.4138460	22.4243326	23.6931905
C	16.7774905	21.4511707	22.8021362
C	15.2769914	21.4889880	23.0549870
O	14.8056348	21.4096016	24.1983627
C	17.2531286	20.0091994	23.0363914
S	16.4431000	18.8024924	21.9128833
N	14.4860883	21.5797227	21.9557149
C	13.0548600	21.4292037	22.0590402
C	12.5789930	19.9659888	22.1149876
O	11.5615364	19.6821078	22.7773192
C	12.3783708	22.1982279	20.9106646
O	13.0223703	21.7490361	19.7083104
C	12.5110681	23.7059622	21.0988081
N	13.2720767	19.0918878	21.3744418
C	12.9154152	17.6839492	21.2417806
C	12.8849928	16.9619904	22.5969874
O	12.0724391	16.0794443	22.8611889
C	13.8760020	17.0012739	20.2447535
O	13.2085430	15.8855920	19.7216895
N	13.8191339	17.4118190	23.4944597
C	13.9422374	16.8718285	24.8366684
C	12.6639928	16.9999904	25.6659856
O	12.4769522	16.3050590	26.6679952
C	15.0624510	17.5960259	25.6080738
C	16.4744397	17.2516724	25.1280828
C	16.8617702	15.7838245	25.3609810
C	17.6189870	15.2112151	24.1736854
N	17.9286554	13.7725267	24.3973765
H	18.4297610	22.3171647	23.6414941
H	17.1352917	22.2055621	24.6537889
H	18.3360533	19.9530364	22.8900281
H	17.0273040	19.7258160	24.0729533
H	16.9949745	21.7280256	21.7587021
H	14.8991878	21.5978865	21.0258497
H	12.7406524	21.8613683	23.0182102
H	11.3109850	21.9114781	20.8961341
H	13.5668327	23.9962460	21.1860865
H	12.0633836	24.2481268	20.2527610
H	11.9859314	24.0256526	22.0110176
H	12.8593330	22.3960852	19.0029249
H	14.1919889	19.3779020	21.0263791
H	11.8995886	17.6015913	20.8317542
H	14.1410240	17.7427629	19.4642217
H	14.8207144	16.7305060	20.7568995
H	13.8311003	15.3403081	19.1497018
H	14.1402325	15.7882027	24.8002023
H	14.5736312	18.0166431	23.1460450
H	14.8896983	18.6815186	25.5193739
H	14.9500647	17.3323902	26.6701771
H	17.1959625	17.9100613	25.6294896
H	16.5590864	17.4837766	24.0546245
H	17.4693109	15.6766121	26.2717143
H	15.9511879	15.1800781	25.5200097
H	17.0386675	15.3159387	23.2462414
H	18.5782628	15.7152216	24.0105829
H	18.6435435	13.6499734	25.2182484
H	17.0749180	13.2436773	24.6073651
N	11.8075425	17.9909791	25.2927802
C	10.5435320	18.1211815	25.9772700
C	9.6159946	16.9249904	25.7139856
O	8.4948763	16.8627707	26.1741981
C	9.8729748	19.4414484	25.5957613
C	10.7046022	20.6351137	26.0643777
C	10.1391647	21.9719551	25.5812785
C	10.9015303	23.1690595	26.1220244
N	10.7826127	23.1893041	27.6510588
H	10.0476327	16.1306916	25.0607185
H	10.7331500	18.0954565	27.0659810
H	9.7667732	19.4910054	24.5011759
H	8.8668505	19.4598336	26.0384583
H	11.7379403	20.5289989	25.6996979
H	10.7544485	20.5832325	27.1732449
H	10.2078056	22.0066196	24.4833939
H	9.0661488	22.0669262	25.8263999
H	11.9761238	23.1024232	25.9043958
H	11.0674299	22.2608091	28.0064573
H	9.8071503	23.3456507	27.9391852
H	11.3762999	23.9085510	28.0842733
H	18.4205237	13.2969952	23.6028884
H	11.8975197	18.4158390	24.3628651
H	10.5152177	24.1338317	25.7720667
Fe	17.9849047	17.9848005	20.5514797
Fe	20.5768398	17.7527421	20.4983462
S	19.4652248	19.7079274	20.4264237
S	19.1450912	16.1471912	21.2081656
O	23.3759471	13.0781040	24.5212271
O	23.2100184	11.9011295	25.0713856

Large model *Cp* Fe… O_2 3.5 Å
255

N	21.3180396	10.3813623	28.6850161
C	21.8147068	11.3113279	29.7376918
C	20.6319942	12.0059966	30.3499915
O	20.3847089	12.0731051	31.5403187
C	22.7079310	12.3419955	29.0123171
C	23.2219992	11.5459153	27.8132692
C	21.9792362	10.7523458	27.4054042
N	29.8973941	16.0201467	24.7530414
C	28.5917908	15.4612931	24.3607538
C	27.8758578	16.5770995	23.6043449
O	27.4603375	16.4739616	22.4672174
C	27.7069154	14.9553701	25.5219776

C	27.9447744	13.5230434	25.9364212
C	29.2312437	12.9932961	26.1293832
C	26.8460124	12.6757224	26.1384662
C	29.4117696	11.6608410	26.5043088
C	27.0214409	11.3473218	26.5292972
C	28.3060751	10.8322953	26.7103159
H	24.0151915	10.8575884	28.1440035
H	23.6101608	12.1877793	27.0154246
H	23.4940762	12.7411492	29.6626496
H	22.0950450	13.1882719	28.6617177
H	22.2031253	9.8484249	26.8227945
H	21.3028903	11.3884103	26.8144260
H	22.3696280	10.7798120	30.5261904
H	19.9559374	12.4677106	29.5831263
H	21.5708543	9.4281397	28.9403090
H	25.8396189	13.0667715	25.9810487
H	26.1467619	10.7146060	26.6766510
H	28.4454712	9.7920543	27.0084079
H	30.4212078	11.2690989	26.6402848
H	30.1050070	13.6294979	25.9850668
H	28.7534791	14.6482029	23.6380002
H	30.6550819	15.3569039	24.6072789
H	29.9035954	16.3078076	25.7314061
H	27.7850169	17.5389510	24.1691503
H	27.8466808	15.6347063	26.3816460
H	26.6460072	15.0689225	25.2453273
N	19.0009069	4.5986342	27.9770563
C	18.4875629	5.9656113	27.9588378
C	19.5609945	7.0299980	27.7839922
O	20.7496008	6.7794191	27.8004374
C	17.7443006	6.2939206	29.2721515
C	16.4959290	5.4347111	29.4385868
C	15.8109336	5.6697297	30.7872162
C	14.5059327	4.8974461	30.8726231
N	13.7949961	5.1749985	32.1859910
H	14.3731129	4.8544684	32.9995756
H	12.8694381	4.6844411	32.2398364
H	13.6189774	6.2005093	32.3032352
H	13.8038725	5.1879301	30.0803819
H	14.6583242	3.8114060	30.8287279
H	16.4831144	5.3664505	31.6066369
H	15.6104722	6.7461858	30.9223527
H	16.7856308	4.3778682	29.3317056
H	15.7880200	5.6595401	28.6229374
H	18.4389377	6.1163161	30.1095682
H	17.4804896	7.3639182	29.2972474
H	17.7685826	6.0852690	27.1282341
H	19.9795615	4.6356841	28.2848407
H	19.0301155	4.2204639	27.0291637
H	19.2014040	8.0850611	27.6944695
N	24.1653139	19.2402185	19.7386477
C	23.2730439	20.0151101	20.5971221
C	23.7999933	21.4469940	20.7309942
O	23.1552363	22.3744659	21.1710676
C	23.0567266	19.4926211	22.0272011
S	22.5340309	17.7374755	22.0701469
Fe	22.2914365	17.0231441	24.2283560
Fe	22.7069936	14.9339958	25.5569928
S	24.3564074	15.9228416	24.3486796
S	21.4492359	14.9705571	23.6095423
C	22.4112741	14.3310684	22.1219127
C	24.6473263	15.0567900	22.7046574
O	19.6528337	18.2402683	24.3813174
N	23.7778149	19.5037527	25.2732601
O	21.5818818	17.1215410	27.1102191
N	19.9779351	13.7618040	26.3632666
O	24.4355100	14.9388258	27.9120903
C	20.7069033	17.7537102	24.3140202
C	23.1971973	18.5606903	24.8872355
C	22.0070163	16.6199146	26.1355977
C	21.0771665	14.1503249	26.1810741
C	23.7328988	14.9092188	26.9871646
N	23.7768757	13.9942945	22.3219472
H	23.8841343	13.1627393	22.9038360
H	24.6014300	15.8678977	21.9639161
H	25.6783879	14.6936091	22.7643189
H	21.8532973	13.4389444	21.8120513
H	22.3160469	15.1093105	21.3506393

H	23.9831760	19.5278259	22.6167128
H	22.3098322	20.1011363	22.5510139
H	24.9576898	18.8708514	20.2707720
H	23.6639827	18.4332016	19.3604183
H	22.2926652	20.0880495	20.0971808
H	24.8569172	21.5760576	20.3771914
N	20.4019828	8.8935304	21.5238069
C	20.4282100	9.8438290	22.6407595
C	19.7739944	11.1869969	22.2599937
O	19.3784802	11.9722618	23.1358270
C	21.8462633	10.1224442	23.1367495
S	22.9376077	10.5394385	21.7125489
N	19.6918187	11.3868859	20.9352263
C	19.0251132	12.5111124	20.3209448
C	17.5669951	12.1609966	19.9869944
O	17.1943131	11.0403139	19.7047744
C	19.7774970	12.9303642	19.0520667
S	18.8827853	14.2044600	18.0804179
N	14.5432665	14.0703256	18.1638651
C	15.2886603	14.3395505	16.9165735
C	15.4209957	13.0529963	16.0979955
O	14.4393775	12.5182669	15.5757374
N	16.6653310	12.4955605	16.0867740
C	16.9851714	11.2164535	15.4602383
C	16.0659955	10.0749972	15.9179955
O	15.6805791	9.1865477	15.1940956
C	18.4475051	10.8741161	15.8617119
C	18.8431639	9.4424604	15.6888058
C	19.2601803	8.6186749	16.7128490
C	18.8329287	8.6390415	14.4924332
N	19.5176411	7.3518669	16.2202032
C	19.2584734	7.3274320	14.8631207
C	18.4758128	8.8775502	13.1541228
C	19.3354433	6.2761341	13.9451951
C	18.5488172	7.8340276	12.2384304
C	18.9735612	6.5471337	12.6286074
N	20.8143014	17.7269901	14.9894345
C	21.1084517	19.1335999	14.8274044
C	22.4139937	19.3509946	14.0919960
O	23.2534836	18.4928284	13.9101377
C	21.2158548	19.9602407	16.1385982
S	19.6099102	20.0917508	17.0072315
H	24.0908806	10.3213976	22.3812866
H	22.2538331	9.2410149	23.6462173
H	21.8212905	10.9610577	23.8413002
H	19.8522005	9.4841457	23.5091801
H	19.7083874	8.1604493	21.6516639
H	21.3234937	8.4838725	21.3637538
H	19.9202666	10.5504286	20.3870804
H	19.0265475	13.3542823	21.0307528
H	20.7788973	13.2875005	19.3233140
H	19.8977336	12.0531834	18.3969265
H	16.8630005	13.0257317	20.0197138
H	19.8426301	6.5669537	16.7678547
H	19.6546897	5.2779556	14.2492239
H	19.0115654	5.7474112	11.8881007
H	18.2611032	8.0069835	11.2010415
H	18.1293142	9.8620710	12.8395050
H	19.3811238	8.8415944	17.7688980
H	19.1146564	11.5521618	15.3047317
H	18.5569619	11.1269154	16.9277301
H	16.8870961	11.2810404	14.3656140
H	15.8162709	10.1062257	17.0108409
H	17.4166807	12.9924911	16.5791283
H	14.7245534	15.0689302	16.3206370
H	16.2612829	14.7767663	17.1790657
H	15.1041823	13.4503727	18.7554140
H	13.7016149	13.5363610	17.9202289
H	21.9652977	19.5140034	16.8045408
H	21.5172966	20.9955268	15.9163555
H	20.3214776	19.6174330	14.2153713
H	22.5801259	20.4051545	13.7496938
H	21.6798781	17.2163815	15.1793697
H	20.1790217	17.5902769	15.7790174
Fe	19.2703758	16.0952012	19.1918139
Fe	19.4946457	18.6680960	18.6708421
S	21.1825894	17.1771728	18.6019255
S	17.5768155	17.4616911	18.6425744

N	17.4002356	22.4462865	23.6892629
C	16.7809669	21.4467564	22.8160107
C	15.2769957	21.4889940	23.0549935
O	14.7964875	21.4194303	24.1951518
C	17.2581692	20.0086421	23.0843778
S	16.4949161	18.7883289	21.9381482
N	14.4918426	21.5722207	21.9486178
C	13.0578701	21.4284175	22.0489908
C	12.5789965	19.9659944	22.1149938
O	11.5542320	19.6849953	22.7663366
C	12.3786236	22.1804257	20.8912678
O	13.0286422	21.7178739	19.6977602
C	12.5019901	23.6927900	21.0541198
N	13.2792377	19.0889380	21.3835851
C	12.9043396	17.6879963	21.2446220
C	12.8849964	16.9619952	22.5969937
O	12.0605896	16.0918527	22.8676786
C	13.8343905	17.0103310	20.2173157
O	13.1714948	15.8719993	19.7328655
N	13.8326344	17.3954561	23.4887197
C	13.9501894	16.8707439	24.8395583
C	12.6639964	16.9999952	25.6659928
O	12.4791213	16.3114121	26.6726317
C	15.0619591	17.6174837	25.6048418
C	16.4872638	17.2603312	25.1719776
C	16.8868735	15.8079358	25.4892471
C	17.5049577	15.1286014	24.2764674
N	17.8402806	13.7029185	24.5858844
H	18.4182101	22.3633802	23.6349244
H	17.1283178	22.2429847	24.6550348
H	18.3469703	19.9555713	22.9771783
H	16.9977171	19.7344239	24.1151019
H	17.0071861	21.7021711	21.7690751
H	14.9125940	21.5861943	21.0226711
H	12.7439382	21.8738516	23.0022564
H	11.3132815	21.8854167	20.8819898
H	13.5540614	23.9873051	21.1675399
H	12.0847878	24.2150582	20.1808581
H	11.9453226	24.0317286	21.9407116
H	12.6861469	22.2183976	18.9386863
H	14.2070092	19.3668393	21.0510695
H	11.8772606	17.6243454	20.8586604
H	14.0443882	17.7506125	19.4179518
H	14.8068714	16.7680796	20.6906832
H	13.7924719	15.3293846	19.1578733
H	14.1586085	15.7878049	24.8195972
H	14.5952949	17.9893282	23.1371267
H	14.8910705	18.6994846	25.4768310
H	14.9323572	17.3908852	26.6740143
H	17.1909251	17.9486160	25.6582978
H	16.5992983	17.4450895	24.0915179
H	17.6029937	15.7709529	26.3230338
H	15.9981239	15.2373254	25.8137414
H	16.8257763	15.1599821	23.4114935
H	18.4448047	15.6060357	23.9752036
H	18.5847044	13.6624285	25.3735987
H	16.9980869	13.1921224	24.8769540
N	11.8071678	17.9867068	25.2846615
C	10.5430687	18.1227651	25.9684916
C	9.6159973	16.9249952	25.7139928
O	8.4925464	16.8687688	26.1693630
C	9.8745254	19.4425795	25.5803815
C	10.7133566	20.6336378	26.0418182
C	10.1512609	21.9764667	25.5719534
C	10.9244821	23.1623409	26.1231820
N	10.7826157	23.1893106	27.6510666
H	10.0501103	16.1227968	25.0722898
H	10.7340831	18.1038717	27.0572145
H	9.7645205	19.4867780	24.4859488
H	8.8696794	19.4656294	26.0262491
H	11.7423992	20.5260270	25.6650855
H	10.7774385	20.5782885	27.1501111
H	10.2172462	22.0214296	24.4741832
H	9.0792062	22.0757826	25.8200300
H	12.0008500	23.0757046	25.9218896
H	11.0197330	22.2484243	28.0109370
H	9.8114685	23.3932060	27.9258367
H	11.4044969	23.8800480	28.0928353
H	18.2833773	13.1907952	23.7868145
H	11.8956965	18.4038415	24.3517090
H	10.5610437	24.1331309	25.7653061
Fe	18.1133862	17.8821254	20.7161721
Fe	20.7377935	17.6177255	20.7035535
S	19.6210626	19.5727041	20.6452659
S	19.2694299	16.0233788	21.3641811
O	23.8482419	11.7837976	24.5451644
O	24.3226197	10.6975239	24.9541018

Large model *Cp* Fe… O_2 2.4 Å

255

N	21.4221017	10.2857138	28.8297168
C	21.8510990	11.2524536	29.8798367
C	20.6319942	12.0059966	30.3499915
O	20.2924698	12.1403432	31.5119706
C	22.8401806	12.2339465	29.2005819
C	23.3469269	11.4485165	27.9874977
C	22.0876632	10.6852896	27.5615527
N	29.9327742	15.8153907	24.7694290
C	28.6050177	15.3625448	24.3261430
C	27.9879921	16.4943954	23.5201934
O	27.4893220	16.3633071	22.4210777
C	27.6200994	14.9394321	25.4510594
C	27.6851476	13.4678165	25.7710386
C	28.8654300	12.8262634	26.1819234
C	26.5334243	12.6904658	25.5989927
C	28.8890169	11.4447775	26.3921010
C	26.5480664	11.3138906	25.8183366
C	27.7306508	10.6834159	26.2103763
H	24.1485200	10.7514810	28.2817876
H	23.7380601	12.1006701	27.2022187
H	23.6218416	12.5722728	29.8894176
H	22.2913517	13.1260080	28.8587184
H	22.2743003	9.7977217	26.9411273
H	21.4190812	11.3549354	27.0047713
H	22.3069695	10.7396775	30.7418200
H	20.0391859	12.4496292	29.5088901
H	21.7396110	9.3544891	29.0928126
H	25.6127472	13.1781539	25.2781852
H	25.6300007	10.7434942	25.6697711
H	27.7471258	9.6049368	26.3762017
H	29.8166282	10.9598150	26.7022806
H	29.7772259	13.4081465	26.3316022
H	28.7341045	14.5220613	23.6297407
H	30.5768635	15.0302202	24.8551303
H	29.8737209	16.2642127	25.6856371
H	28.0290691	17.4975525	24.0208030
H	27.8210639	15.5592190	26.3411975
H	26.5898398	15.1823484	25.1435163
N	19.0115880	4.6000510	27.9708840
C	18.4891284	5.9644118	27.9700067
C	19.5609945	7.0299980	27.7839922
O	20.7458290	6.7647647	27.7345068
C	17.7603716	6.2818960	29.2937715
C	16.5151857	5.4189316	29.4607989
C	15.8269760	5.6465083	30.8088048
C	14.5048404	4.9020170	30.8713780
N	13.7949961	5.1749985	32.1859910
H	14.3682517	4.8395120	32.9963225
H	12.8632666	4.6967970	32.2341103
H	13.6330910	6.2013368	32.3122614
H	13.8148141	5.2232264	30.0803396
H	14.6313116	3.8134203	30.8093249
H	16.4849861	5.3144861	31.6286109
H	15.6466794	6.7245230	30.9602618
H	16.8071727	4.3629733	29.3510317
H	15.8067397	5.6453553	28.6460382
H	18.4599034	6.0997131	30.1258406
H	17.4931253	7.3508377	29.3290145
H	17.7567152	6.0867387	27.1509581
H	20.0098376	4.6498397	28.2088136
H	18.9792251	4.2074698	27.0292108
H	19.2113366	8.0917436	27.7650414
N	24.1022815	19.2621486	19.6829362
C	23.2156497	20.0449598	20.5406992
C	23.7999933	21.4469940	20.7309942

O	23.1965401	22.3807473	21.2144561
C	22.9335404	19.4861775	21.9462315
S	22.3442753	17.7570145	21.8793821
Fe	22.0634780	16.8764548	23.9556641
Fe	22.5872937	14.7190959	25.0846930
S	24.1824813	15.8957553	23.9583560
S	21.2945801	14.8312750	23.1995121
C	22.2888351	14.3305546	21.6694278
C	24.4930017	15.1656848	22.2585889
O	19.4768720	18.1750651	24.2194193
N	23.3628444	19.3555383	25.2350432
O	21.3038769	16.7141902	26.8088215
N	20.2197483	13.3595712	26.4473391
O	24.2714741	14.8538202	27.4746466
C	20.4912546	17.6241352	24.0884116
C	22.8768808	18.4030881	24.7536760
C	21.7630933	16.3240890	25.7999167
C	21.1716055	13.8342147	25.9369961
C	23.6120950	14.7719276	26.5252285
N	23.6766911	14.0748118	21.8311772
H	23.8297190	13.2336969	22.3899602
H	24.3745419	16.0168459	21.5728804
H	25.5483801	14.8715240	22.2750374
H	21.7972792	13.4155695	21.3181944
H	22.1255240	15.1418778	20.9457418
H	23.8443409	19.4564204	22.5586501
H	22.2025397	20.1061093	22.4791361
H	24.8717089	18.8605074	20.2235185
H	23.5928296	18.4691145	19.2851823
H	22.2545951	20.1720382	20.0148739
H	24.8583368	21.5462825	20.3728611
N	20.4615593	8.9465737	21.3932859
C	20.3476022	9.7954550	22.5907814
C	19.7739944	11.1869969	22.2599937
O	19.4345547	11.9497476	23.1715923
C	21.6750989	9.9881046	23.3237803
S	22.9373913	10.5019672	22.0830820
N	19.6922407	11.4339696	20.9417628
C	19.0103164	12.5566456	20.3378401
C	17.5669951	12.1609966	19.9869944
O	17.2445296	11.0306165	19.6832432
C	19.7503791	12.9950814	19.0668466
S	18.8039056	14.2058527	18.0628472
N	14.5578043	14.0880683	18.1681295
C	15.2760952	14.3474573	16.9027377
C	15.4209957	13.0529963	16.0979955
O	14.4405378	12.5109869	15.5791873
N	16.6659723	12.4985027	16.0849142
C	16.9853865	11.2170747	15.4611671
C	16.0659955	10.0749972	15.9179955
O	15.6803659	9.1883227	15.1916730
C	18.4473571	10.8745377	15.8643162
C	18.8409042	9.4413379	15.6968967
C	19.2649949	8.6249890	16.7235744
C	18.8211850	8.6307782	14.5058059
N	19.5190551	7.3549443	16.2371796
C	19.2490802	7.3209448	14.8823380
C	18.4502700	8.8587430	13.1705704
C	19.3143533	6.2628213	13.9728258
C	18.5100149	7.8071736	12.2618255
C	18.9374789	6.5236728	12.6574554
N	20.7365389	17.7590831	14.8885923
C	21.0488574	19.1598802	14.7122750
C	22.4139937	19.3509946	14.0919960
O	23.2617166	18.4852730	14.0120008
C	21.0514639	20.0293119	16.0002457
S	19.3907981	20.1053126	16.7650830
H	23.9708103	10.4931151	22.9559108
H	21.9944596	9.0481741	23.7927375
H	21.5694333	10.7660486	24.0905569
H	19.6290456	9.3702531	23.3102808
H	19.8931766	8.1056161	21.4704549
H	21.4391915	8.6822017	21.2392336
H	19.9009093	10.6018416	20.3750150
H	18.9872242	13.3959628	21.0506262
H	20.7314286	13.4062618	19.3323138
H	19.9144120	12.1142524	18.4262804
H	16.8235365	12.9917971	20.0354814
H	19.8456678	6.5717666	16.7864505
H	19.6332679	5.2664658	14.2819329
H	18.9660248	5.7174755	11.9234054
H	18.2066623	7.9717059	11.2277173
H	18.0995619	9.8411339	12.8535320
H	19.3932709	8.8571280	17.7767796
H	19.1155205	11.5473911	15.3023162
H	18.5589953	11.1308736	16.9290335
H	16.8891155	11.2797554	14.3662602
H	15.8173447	10.1035549	17.0113705
H	17.4153966	12.9928557	16.5826950
H	14.6840572	15.0531808	16.3052066
H	16.2413313	14.8122175	17.1426153
H	15.1529274	13.5209258	18.7780350
H	13.7369191	13.5092977	17.9589443
H	21.7757963	19.6243772	16.7178478
H	21.3322565	21.0690196	15.7675269
H	20.3189503	19.6196132	14.0169541
H	22.6140699	20.3912254	13.7248725
H	21.5717255	17.2507208	15.1864324
H	20.0239107	17.6484961	15.6143652
Fe	19.1107571	16.1621504	19.0692050
Fe	19.3078056	18.7341728	18.4778654
S	21.0086650	17.2463273	18.4344510
S	17.3967336	17.5108382	18.5027716
N	17.4093285	22.4250851	23.7015310
C	16.7803713	21.4444380	22.8138422
C	15.2769957	21.4889940	23.0549935
O	14.7994338	21.4181240	24.1961700
C	17.2544663	20.0001897	23.0530335
S	16.4365802	18.7955981	21.9319988
N	14.4899234	21.5829779	21.9500843
C	13.0562303	21.4315625	22.0517656
C	12.5789965	19.9659944	22.1149938
O	11.5591219	19.6846253	22.7749136
C	12.3688307	22.1951567	20.9060239
O	13.0045099	21.7390812	19.7030910
C	12.4988641	23.7053801	21.0835719
N	13.2732022	19.0897082	21.3772054
C	12.9047484	17.6852559	21.2440603
C	12.8849964	16.9619952	22.5969937
O	12.0550509	16.0958531	22.8665254
C	13.8382043	17.0070904	20.2186828
O	13.1579863	15.9008936	19.6866375
N	13.8322349	17.3857907	23.4901581
C	13.9415352	16.8357139	24.8302655
C	12.6639964	16.9999952	25.6659928
O	12.4784705	16.3249211	26.6821713
C	15.1010347	17.5110183	25.5880949
C	16.4955051	17.1050021	25.1052995
C	16.8866738	15.6629150	25.4555470
C	17.7548964	15.0589566	24.3639345
N	18.1654313	13.6675651	24.6912410
H	18.4270472	22.3473853	23.6321810
H	17.1484910	22.2034598	24.6661691
H	18.3369953	19.9387302	22.9032403
H	17.0305474	19.7140382	24.0894077
H	17.0054501	21.7181911	21.7714129
H	14.9121952	21.5752555	21.0244310
H	12.7450773	21.8714174	23.0083345
H	11.3009213	21.9062268	20.9062782
H	13.5528340	24.0005913	21.1779630
H	12.0578726	24.2409755	20.2300042
H	11.9628827	24.0291814	21.9876978
H	12.7237372	22.3024297	18.9616166
H	14.1967566	19.3683156	21.0360714
H	11.8806993	17.6128051	20.8534153
H	14.0920054	17.7633308	19.4475260
H	14.7908232	16.7217143	20.7061102
H	13.7825625	15.3475334	19.1239178
H	14.0986242	15.7454647	24.7853657
H	14.6032251	17.9725564	23.1420402
H	14.9778054	18.6019186	25.4870775
H	14.9871305	17.2662885	26.6540884
H	17.2381019	17.7868595	25.5392623
H	16.5665677	17.2529394	24.0154025
H	17.4287707	15.6381607	26.4131993
H	15.9828410	15.0415535	25.5875874

H	17.2271111	15.0432369	23.3990380
H	18.6773759	15.6284890	24.2042868
H	18.8744899	13.6057105	25.5057973
H	17.3515347	13.0855117	24.9155444
N	11.8092934	17.9868802	25.2804054
C	10.5464481	18.1224569	25.9672887
C	9.6159973	16.9249952	25.7139928
O	8.5012006	16.8627161	26.1888635
C	9.8750228	19.4416635	25.5825377
C	10.7062422	20.6354659	26.0513961
C	10.1399205	21.9743670	25.5757813
C	10.9027091	23.1691569	26.1216345
N	10.7825970	23.1892935	27.6510922
H	10.0408843	16.1331027	25.0528471
H	10.7404950	18.1029221	27.0551146
H	9.7687837	19.4899766	24.4881608
H	8.8685855	19.4604977	26.0252874
H	11.7380623	20.5296229	25.6817732
H	10.7597844	20.5806749	27.1599903
H	10.2098291	22.0159206	24.4781518
H	9.0665003	22.0681241	25.8207778
H	11.9778842	23.1012255	25.9068480
H	11.0690875	22.2605765	28.0056554
H	9.8063638	23.3437652	27.9387518
H	11.3760414	23.9092520	28.0840765
H	18.6770949	13.1975343	23.9031614
H	11.8937282	18.3997999	24.3452045
H	10.5177586	24.1341939	25.7716566
Fe	17.9674806	17.9808259	20.5580262
Fe	20.5580527	17.7379228	20.5156163
S	19.4553258	19.6966741	20.4299155
S	19.1162867	16.1422663	21.2326558
O	23.6635238	12.6819010	24.4127090
O	23.4969362	11.5495829	24.9910271
